# Supplementary material for: The association between routine immunisation and COVID-19 vaccination in small Island developing states
Source: PLoS One. 2025 Jul 8;20(7):e0317327. doi: 10.1371/journal.pone.0317327 (PMC12237071; doi:10.1371/journal.pone.0317327)
Supplement: S1 Appendix — (PDF) [file pone.0317327.s001.pdf]

S1: Spearman correlations between COVID-19 vaccination coverage and 5-year (2015–2019) mean annual coverage of routine immunisations

Vaccine	N	June 2021			December 2021			June 2022			December 2022		
		rho	p-value	95% CI	rho	p-value	95% CI	rho	p-value	95% CI	rho	p-value	95% CI
**Coverage of first dose of COVID-19 vaccination**													
BCG	42	0.335	0.032	(-0.007 to 0.63)	0.279	0.074	(-0.067 to 0.595)	0.257	0.1	(-0.069 to 0.604)	0.231	0.142	(-0.098 to 0.557)
DTP1	51	0.334	0.018	(0.073 to 0.567)	0.35	0.012	(0.083 to 0.59)	0.334	0.017	(0.031 to 0.582)	0.314	0.025	(0.007 to 0.559)
DTP3	52	0.264	0.061	(-0.009 to 0.515)	0.348	0.012	(0.06 to 0.597)	0.258	0.064	(-0.068 to 0.541)	0.222	0.114	(-0.083 to 0.508)
HepB birth dose	40	0.367	0.02	(0.024 to 0.66)	0.374	0.017	(-0.004 to 0.685)	**0.425**	**0.006**	**(0.056 to 0.73)**	**0.402**	**0.01**	**(0.05 to 0.693)**
MCV1	52	0.338	0.015	(0.069 to 0.566)	0.399	0.003	(0.116 to 0.653)	0.341	0.013	(0.035 to 0.622)	0.304	0.028	(0.004 to 0.588)
MCV2	47	0.245	0.101	(-0.054 to 0.491)	0.377	0.009	(0.077 to 0.611)	0.338	0.02	(0.036 to 0.592)	0.317	0.03	(0.004 to 0.573)
**Full coverage of primary series of COVID-19 vaccination**													
BCG	42	0.309	0.049	(-0.04 to 0.613)	0.294	0.059	(-0.051 to 0.599)	0.242	0.123	(-0.108 to 0.566)	0.230	0.143	(-0.104 to 0.559)
DTP1	51	0.311	0.028	(0.041 to 0.566)	0.392	0.004	(0.148 to 0.631)	0.360	0.009	(0.078 to 0.602)	0.345	0.013	(0.057 to 0.586)
DTP3	52	0.255	0.071	(-0.038 to 0.497)	0.378	0.006	(0.091 to 0.62)	0.311	0.025	(0.011 to 0.6)	0.250	0.074	(-0.047 to 0.535)
HepB birth dose	40	0.279	0.081	(-0.047 to 0.586)	0.369	0.019	(0.026 to 0.659)	**0.421**	**0.007**	**(0.072 to 0.721)**	**0.438**	**0.005**	**(0.098 to 0.715)**
MCV1	52	0.267	0.058	(-0.013 to 0.519)	**0.420**	**0.002**	**(0.145 to 0.664)**	0.373	0.007	(0.08 to 0.658)	0.334	0.016	(0.039 to 0.625)
MCV2	47	0.178	0.236	(-0.132 to 0.434)	0.393	0.006	(0.103 to 0.628)	0.359	0.013	(0.043 to 0.621)	0.349	0.016	(0.033 to 0.609)

BCG: Bacillus Calmette–Guérin; CI: Confidence interval; DTP: Diphtheria-tetanus-pertussis; HepB: Hepatitis B; MCV: Measles-containing vaccine
Bolded numbers show |r|>0.4 and p<0.05
