# Supplementary material for: The association between routine immunisation and COVID-19 vaccination in small Island developing states
Source: PLoS One. 2025 Jul 8;20(7):e0317327. doi: 10.1371/journal.pone.0317327 (PMC12237071; doi:10.1371/journal.pone.0317327)
Supplement: S2 Appendix — (PDF) [file pone.0317327.s002.pdf]

S2: Spearman correlations between COVID-19 vaccination coverage and dropout of annual coverage (5-year mean, 2015–2019) of routine immunisations

Vaccine	June 2021			December 2021			June 2022			December 2022		
	rho	p-value	95% CI	rho	p-value	95% CI	rho	p-value	95% CI	rho	p-value	95% CI
**Coverage of first dose of COVID-19 vaccination**												
DTP1-DTP3	-0.266	0.061	(-0.488 to 0.009)	**-0.418**	0.002	(-0.639 to -0.133)	-0.285	0.043	(-0.536 to 0.003)	-0.250	0.077	(-0.501 to 0.028)
DTP1-MCV1	-0.388	0.005	(-0.589 to -0.142)	**-0.461**	0.001	(-0.675 to -0.185)	-0.346	0.013	(-0.569 to -0.076)	-0.297	0.034	(-0.525 to -0.028)
DTP1-MCV2	-0.223	0.142	(-0.496 to 0.065)	**-0.416**	0.004	(-0.642 to -0.118)	-0.349	0.017	(-0.599 to -0.046)	-0.326	0.027	(-0.576 to -0.028)
MCV1-MCV2	-0.240	0.108	(-0.491 to 0.047)	**-0.420**	0.003	(-0.627 to -0.142)	-0.365	0.012	(-0.589 to -0.071)	-0.351	0.016	(-0.577 to -0.06)
BCG-MCV1	-0.291	0.064	(-0.56 to 0.047)	**-0.451**	0.003	(-0.687 to -0.162)	-0.304	0.050	(-0.573 to 0.017)	-0.262	0.093	(-0.543 to 0.065)
**Full coverage of primary series of COVID-19 vaccination**												
DTP1-DTP3	-0.240	0.093	(-0.474 to 0.055)	**-0.412**	0.003	(-0.629 to -0.143)	-0.338	0.015	(-0.581 to -0.055)	-0.265	0.060	(-0.518 to 0.014)
DTP1-MCV1	-0.304	0.032	(-0.539 to -0.031)	**-0.463**	0.001	(-0.673 to -0.198)	-0.384	0.005	(-0.611 to -0.111)	-0.322	0.021	(-0.543 to -0.05)
DTP1-MCV2	-0.158	0.299	(-0.44 to 0.14)	**-0.408**	0.005	(-0.632 to -0.113)	-0.372	0.011	(-0.62 to -0.048)	-0.363	0.013	(-0.608 to -0.056)
MCV1-MCV2	-0.212	0.156	(-0.48 to 0.092)	**-0.421**	0.003	(-0.638 to -0.138)	-0.380	0.008	(-0.605 to -0.084)	-0.384	0.008	(-0.61 to -0.097)
BCG-MCV1	-0.244	0.125	(-0.523 to 0.105)	**-0.451**	0.003	(-0.687 to -0.149)	-0.355	0.021	(-0.617 to -0.04)	-0.301	0.053	(-0.572 to 0.013)

BCG: Bacillus Calmette–Guérin; CI: Confidence interval; DTP: Diphtheria-tetanus-pertussis; HepB: Hepatitis B; MCV: Measles-containing vaccine
Bolded numbers show |r|>0.4 and p<0.05
