# Supplementary material for: The association between routine immunisation and COVID-19 vaccination in small Island developing states
Source: PLoS One. 2025 Jul 8;20(7):e0317327. doi: 10.1371/journal.pone.0317327 (PMC12237071; doi:10.1371/journal.pone.0317327)

S3: COVID-19 vaccination coverage by new vaccine introductions

Note: Figures show COVID-19 vaccination by new vaccine introduction status: 1) introduced <5 years, 2) introduced ≥5 years, 3) not introduced. Dots represent COVID-19 vaccination coverage in each country, rounded to the nearest coverage point (e.g. 91.7% is rounded to 92%). The red marker represents mean COVID-19 vaccination coverage for the vaccine introduction category.
(graphs from next page)

3A. Time since new vaccine introduction: Pneumococcal conjugate vaccine (PCV)

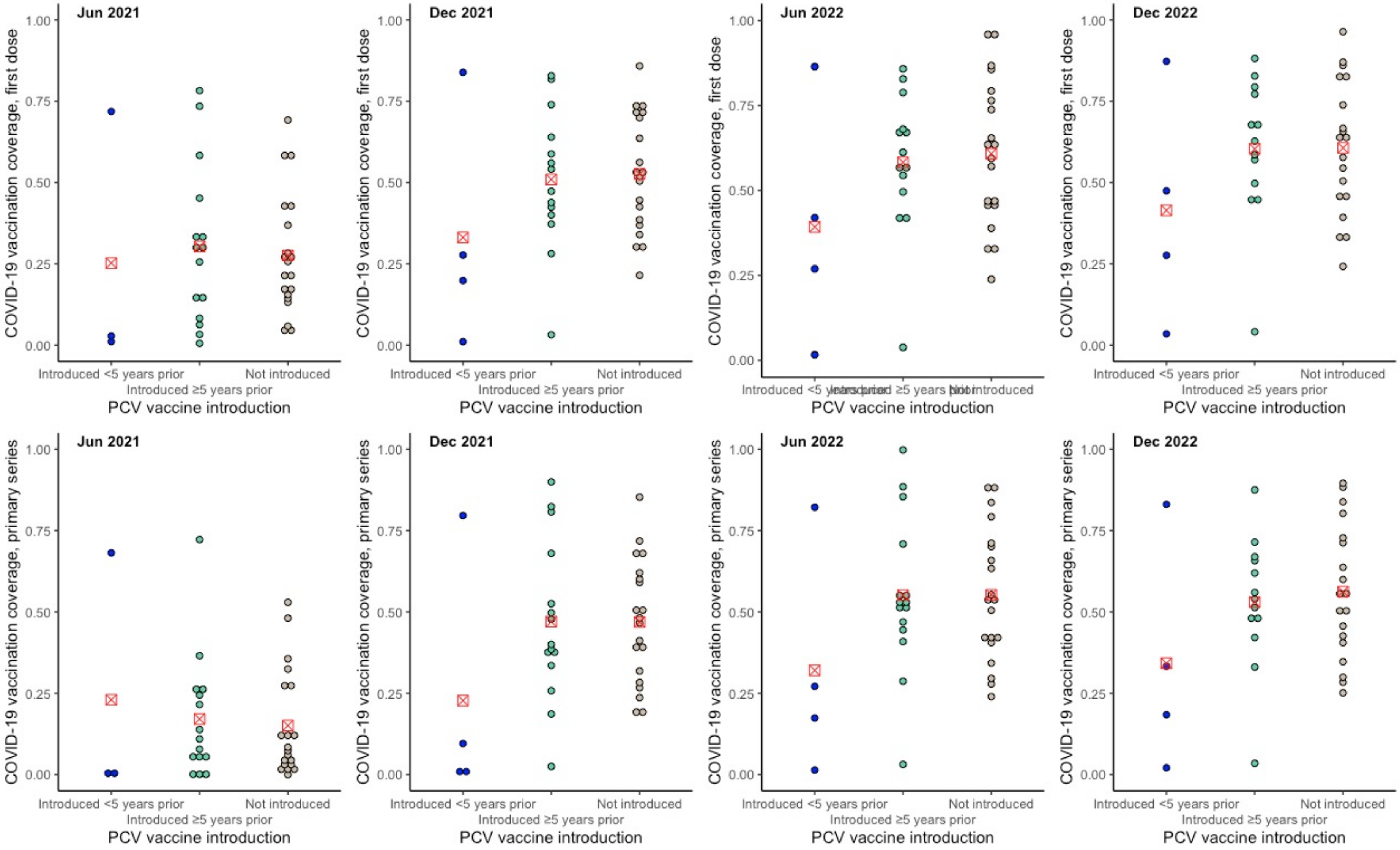

3B. Time since new vaccine introduction: Rotavirus vaccine

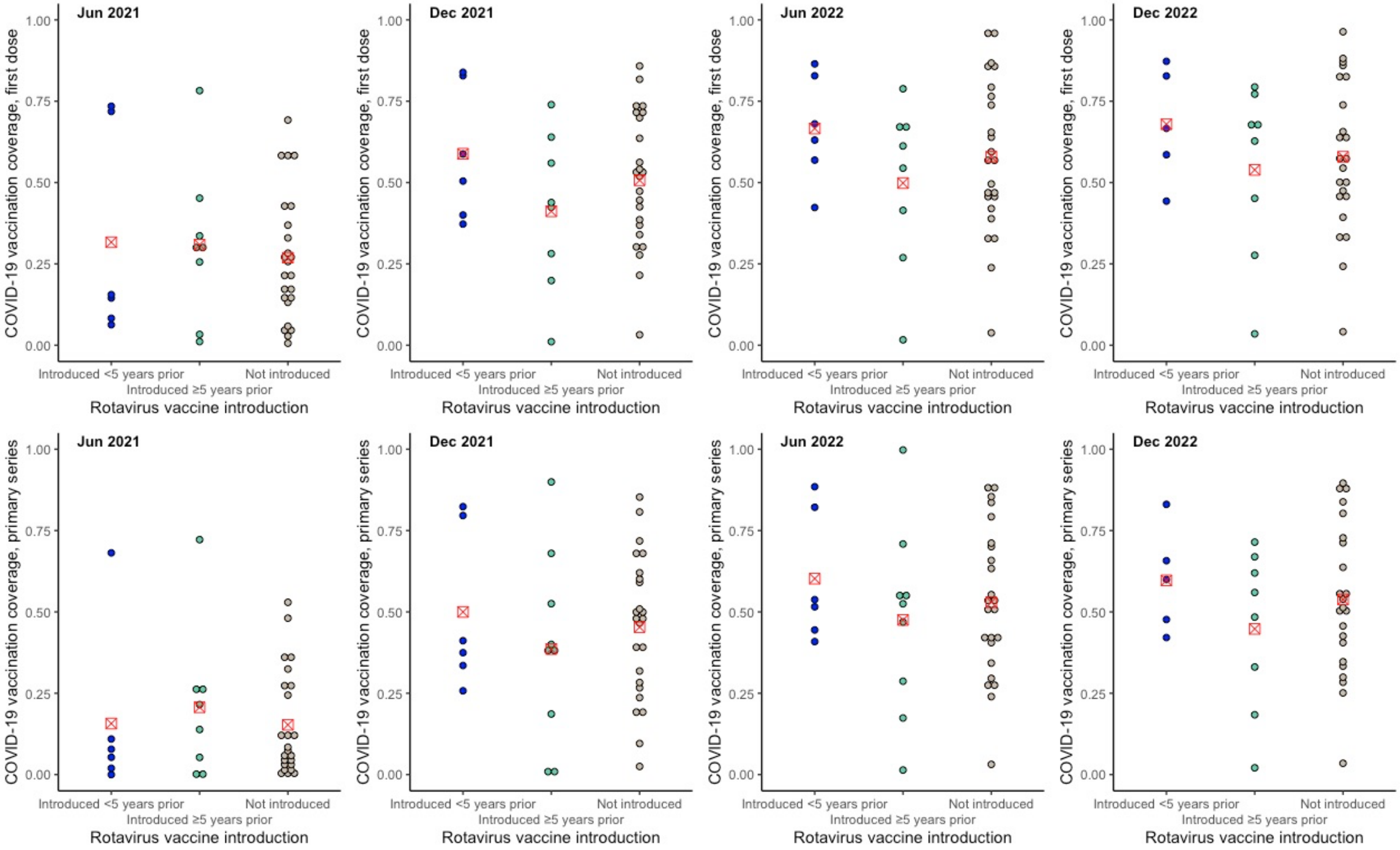

Supplement: S3 Appendix — (PDF) [file pone.0317327.s003.pdf]
