# Supplementary material for: The association between routine immunisation and COVID-19 vaccination in small Island developing states
Source: PLoS One. 2025 Jul 8;20(7):e0317327. doi: 10.1371/journal.pone.0317327 (PMC12237071; doi:10.1371/journal.pone.0317327)
Supplement: S4 Appendix — (PDF) [file pone.0317327.s004.pdf]

S4: Spearman correlations between COVID-19 vaccination coverage and density of health resources (workforce and hospital beds)

Vaccine	June 2021			December 2021			June 2022			December 2022		
	rho	p-value	95% CI	rho	p-value	95% CI	rho	p-value	95% CI	rho	p-value	95% CI
**Coverage of first dose of COVID-19 vaccination**												
Physicians per 1,000*	**0.905**	**<0.001**	**(0.729 to 0.964)**	**0.759**	**<0.001**	**(0.452 to 0.895)**	**0.643**	**0.001**	**(0.319 to 0.846)**	**0.608**	**0.002**	**(0.252 to 0.815)**
Nurses and midwives per 1,000	**0.598**	**0.002**	**(0.178 to 0.879)**	**0.517**	**0.011**	**(0.089 to 0.792)**	0.389	0.061	(-0.057 to 0.694)	0.360	0.085	(-0.089 to 0.662)
Hospital beds per 10,000	**0.581**	**<0.001**	**(0.305 to 0.783)**	0.312	0.060	(-0.048 to 0.612)	0.290	0.081	(-0.077 to 0.598)	0.297	0.075	(-0.069 to 0.589)
**Full coverage of primary series of COVID-19 vaccination**												
Physicians per 1,000*	**0.897**	**<0.001**	**(0.745 to 0.951)**	**0.785**	**<0.001**	**(0.519 to 0.899)**	**0.654**	**0.001**	**(0.306 to 0.849)**	**0.625**	**0.001**	**(0.271 to 0.832)**
Nurses and midwives per 1,000	**0.630**	**0.001**	**(0.237 to 0.850)**	**0.605**	**0.002**	**(0.209 to 0.850)**	0.429	0.038	(-0.033 to 0.736)	0.430	0.037	(-0.009 to 0.730)
Hospital beds per 10,000	**0.641**	**<0.001**	**(0.369 to 0.818)**	0.370	0.024	(0.006 to 0.665)	0.314	0.059	(-0.056 to 0.607)	0.315	0.057	(-0.057 to 0.595)

Bolded values show moderate correlations i.e. r≥0.4 and <0.7
Shaded and bolded values show strong correlations i.e. r≥0.7
* One country was an outlier and excluded from this analysis. Correlation values were strong in a sensitivity analysis that included the outlier country.
