# Supplementary material for: The association between routine immunisation and COVID-19 vaccination in small Island developing states
Source: PLoS One. 2025 Jul 8;20(7):e0317327. doi: 10.1371/journal.pone.0317327 (PMC12237071; doi:10.1371/journal.pone.0317327)

S5: Scatterplots of COVID-19 vaccination coverage and workforce density

*5A. Density of physicians (per 10,000) and coverage of first dose of COVID-19 vaccination**

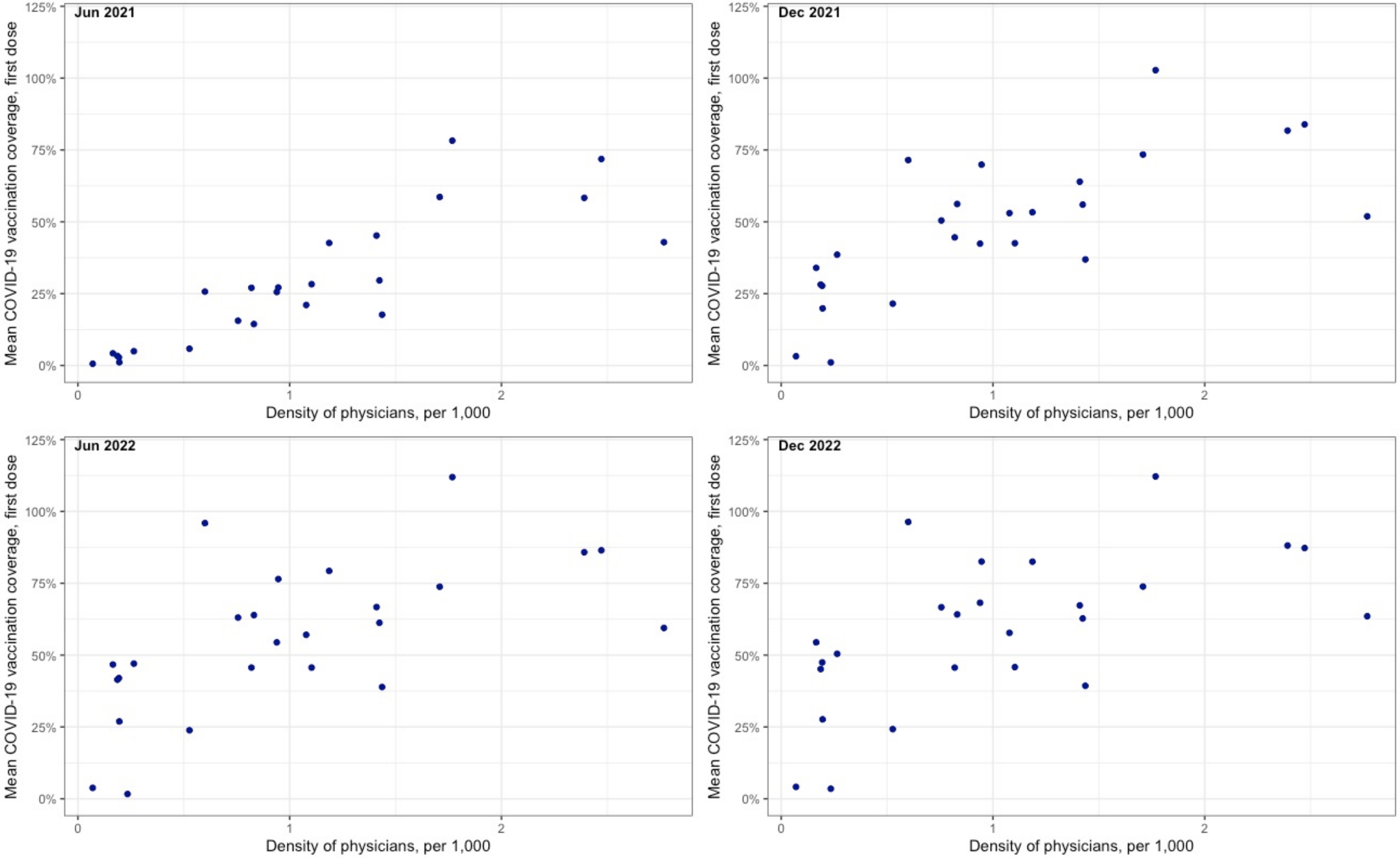

* One country was an outlier and excluded

*5B. Density of physicians (per 10,000) and coverage of primary series of COVID-19 vaccination**

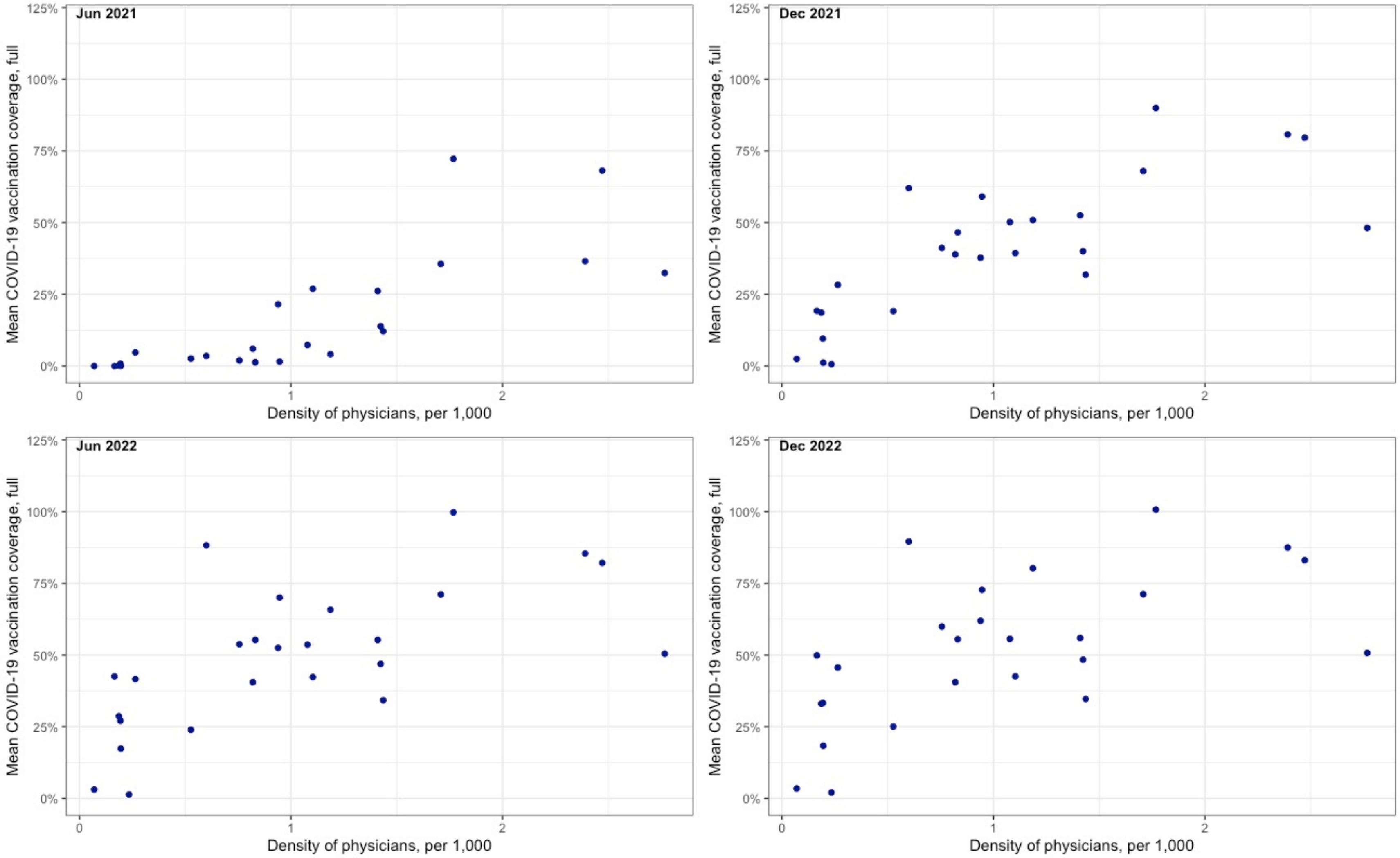

* One country was an outlier and excluded

5C. Density of nurses and midwives (per 10,000) and first dose of COVID-19 vaccination

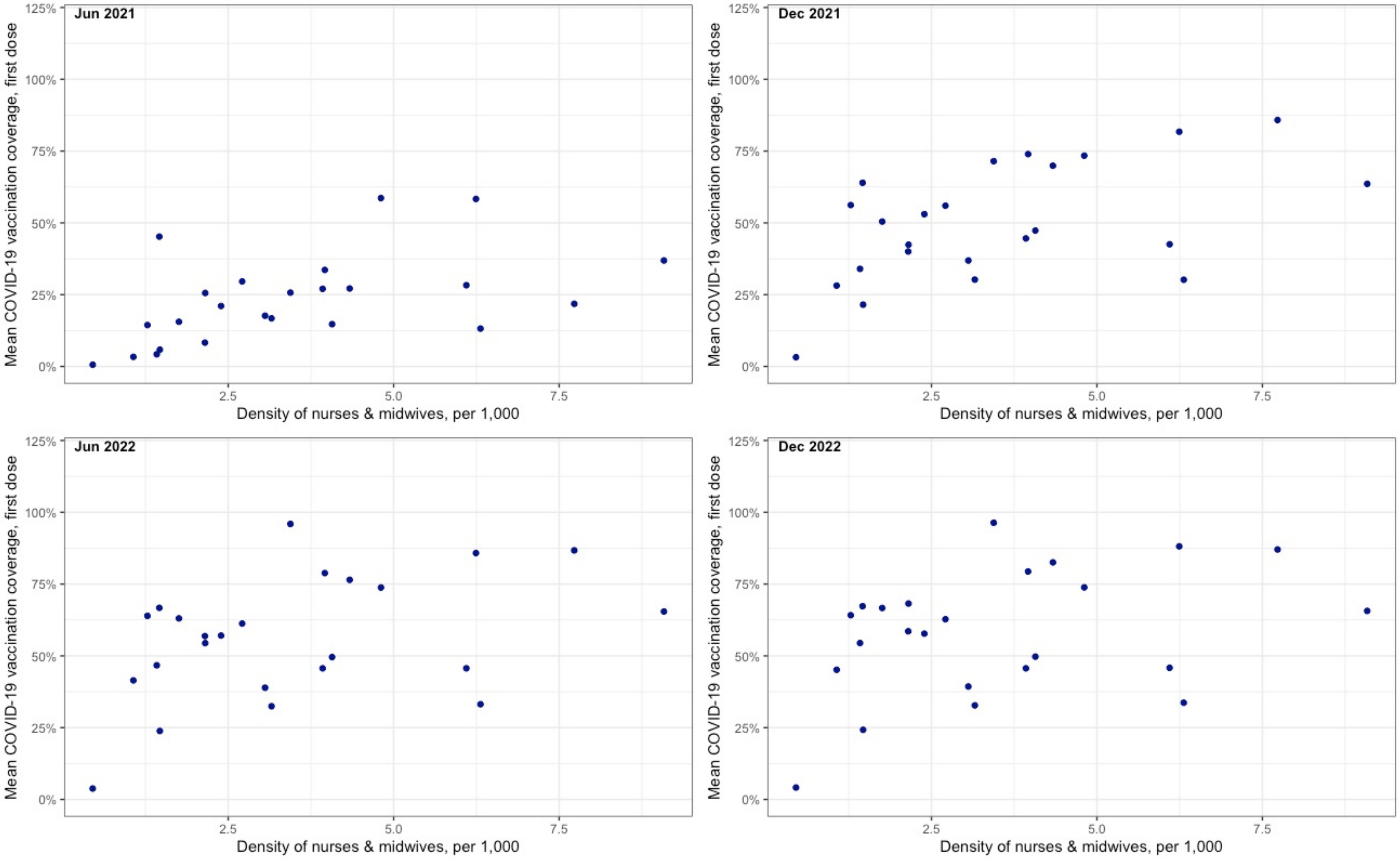

5D. Density of nurses and midwives (per 10,000) and first dose of COVID-19 vaccination

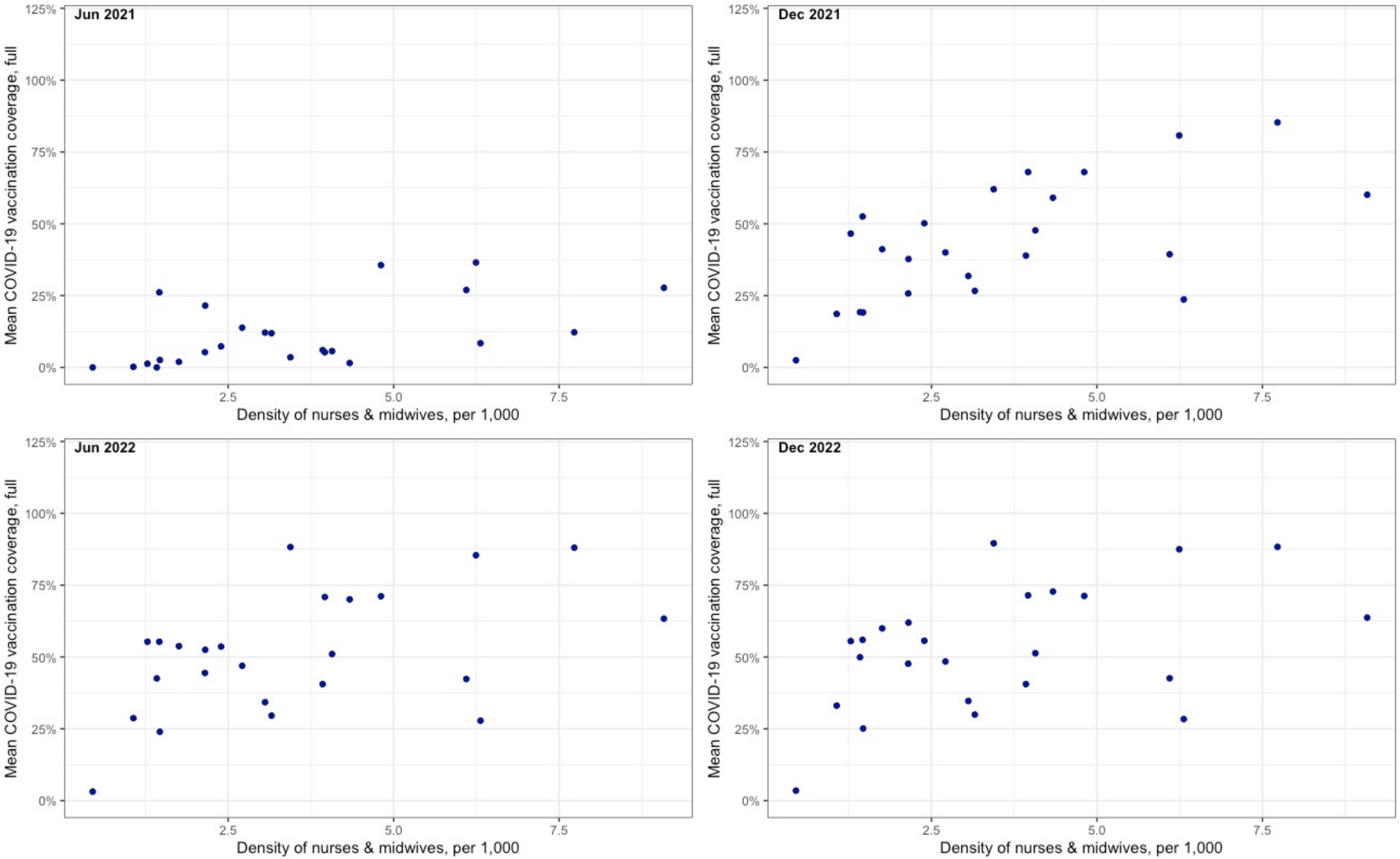

Supplement: S5 Appendix — (PDF) [file pone.0317327.s005.pdf]
