# Supplementary material for: The association between routine immunisation and COVID-19 vaccination in small Island developing states
Source: PLoS One. 2025 Jul 8;20(7):e0317327. doi: 10.1371/journal.pone.0317327 (PMC12237071; doi:10.1371/journal.pone.0317327)
Supplement: S6 Appendix — (PDF) [file pone.0317327.s006.pdf]

S6: COVID-19 vaccination coverage at four timepoints in 2021 and 2022, by health system variables included in the study

Variable	N	Coverage of first dose of COVID-19 vaccine				Coverage of primary series of COVID-19 vaccine			
		Jun-21	Dec-21	Jun-22	Dec-22	Jun-21	Dec-21	Jun-22	Dec-22
**AEFI surveillance system is established**									
Yes	23	28.4%	50.0%	57.7%	59.4%	19.2%	43.4%	51.3%	53.7%
No	3	56.6%	74.8%	85.0%	99.9%	20.2%	69.8%	79.3%	92.8%
N/A (missing or not reported)	31	36.9%	59.2%	64.8%	67.2%	28.0%	53.0%	60.0%	62.1%
**AEFI system is functioning (i.e. Yes to established and reported ≥1 AEFI case)**									
Established and reported ≥1 AEFI case	11	30.4%	51.3%	55.8%	56.8%	20.9%	45.3%	51.5%	52.8%
Established, no reports	12	26.4%	48.6%	59.6%	62.2%	17.6%	41.4%	51.0%	54.7%
No	3	56.6%	74.8%	85.0%	99.9%	20.2%	69.8%	79.3%	92.8%
N/A (missing or not reported)	31	36.9%	59.2%	64.8%	67.2%	28.0%	53.0%	60.0%	62.1%
**NITAG exists – Country meets NITAG functionality criteria (strict definition) ***									
No	18	31.9%	55.7%	63.9%	65.7%	23.4%	48.5%	57.6%	59.3%
Yes	14	30.5%	50.4%	54.2%	55.1%	22.5%	46.9%	51.0%	52.1%
N/A (missing or not reported)	23	38.9%	60.3%	67.4%	72.2%	25.3%	52.8%	61.1%	65.9%
**NITAG exists – Country meets NITAG functionality criteria (permissive definition) ***									
Yes	16	33.0%	53.1%	57.0%	58.0%	25.5%	49.7%	53.9%	55.0%
No	16	29.7%	53.5%	62.3%	64.3%	20.6%	46.0%	55.5%	57.3%
N/A (missing or not reported)	23	38.9%	60.3%	67.4%	72.2%	25.3%	52.8%	61.1%	65.9%

AEFI = Adverse event following immunisation; NITAG = National Immunisation Technical Advisory Group

* Criteria for NITAG functionality included:

A. The advisory group has formal written Terms of References
B. There are legislative or administrative basis for the advisory group
C. Members represent at least five of the following expertise areas: 1) paediatricians, 2) public health experts, 3) infectious disease experts; 4) epidemiology experts; 5) immunology experts, 6) other experts
D. The advisory group meets at least once per annum
E. The agenda and background documents are distributed (at least 1 week) prior to the meeting(s)
F. Members of the advisory group are required to disclose conflicts of interest.

The following definitions were applied:

- Strict definition: must meet all 6 NITAG functionality criteria (for criteria C, at least 5 areas of expertise must be represented)
- Permission definition: must meet criteria B (has legislative basis), D (meets at least once per annum) and has at least 4 expertise areas represented (i.e. C)
