# Supplementary material for: The association between routine immunisation and COVID-19 vaccination in small Island developing states
Source: PLoS One. 2025 Jul 8;20(7):e0317327. doi: 10.1371/journal.pone.0317327 (PMC12237071; doi:10.1371/journal.pone.0317327)

S7: Scatterplots of COVID-19 vaccination coverage and health system variables included in the study

7A. Proportion of births registered

Coverage of first dose of COVID-19 vaccination

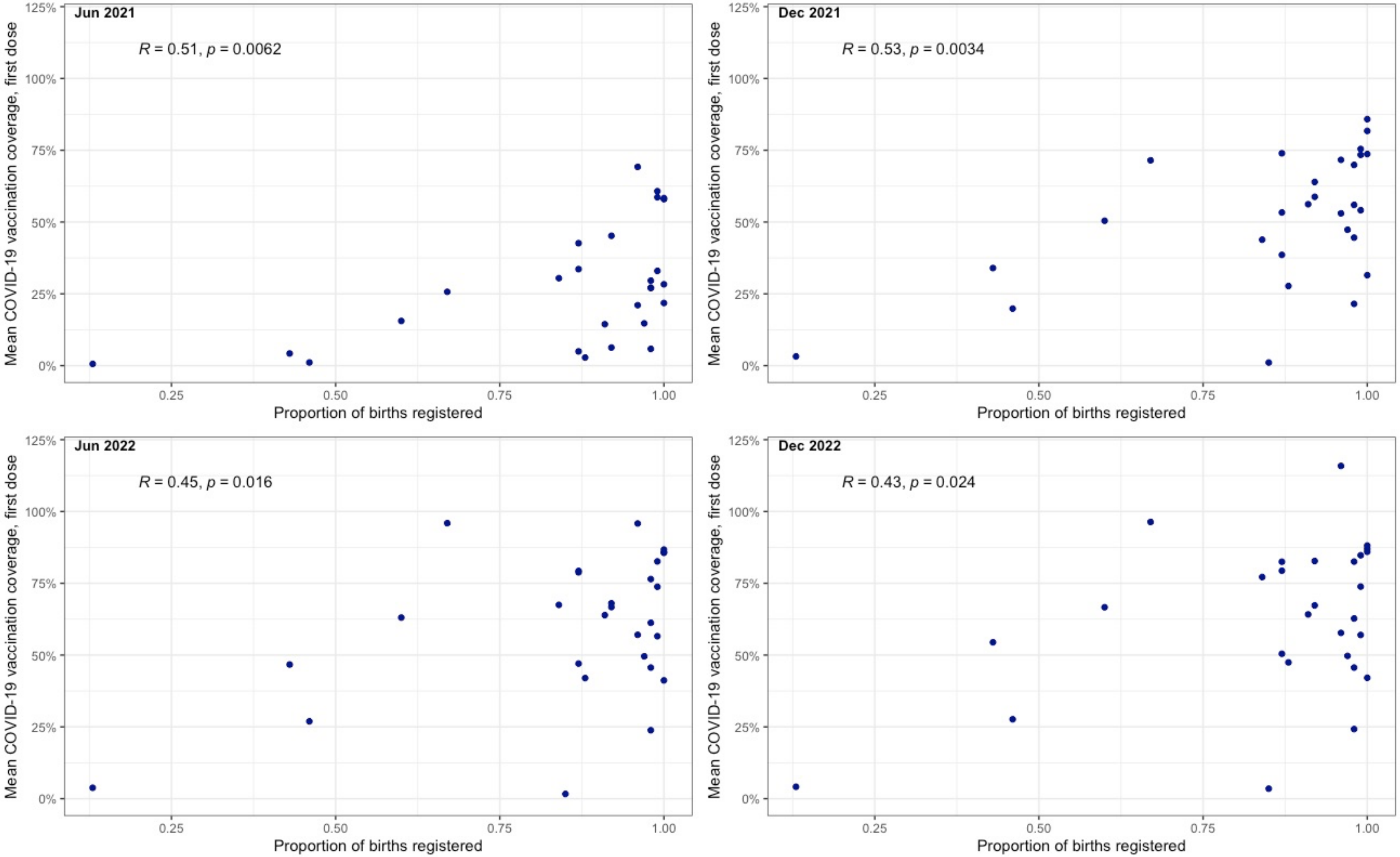

Coverage of primary series of COVID-19 vaccination

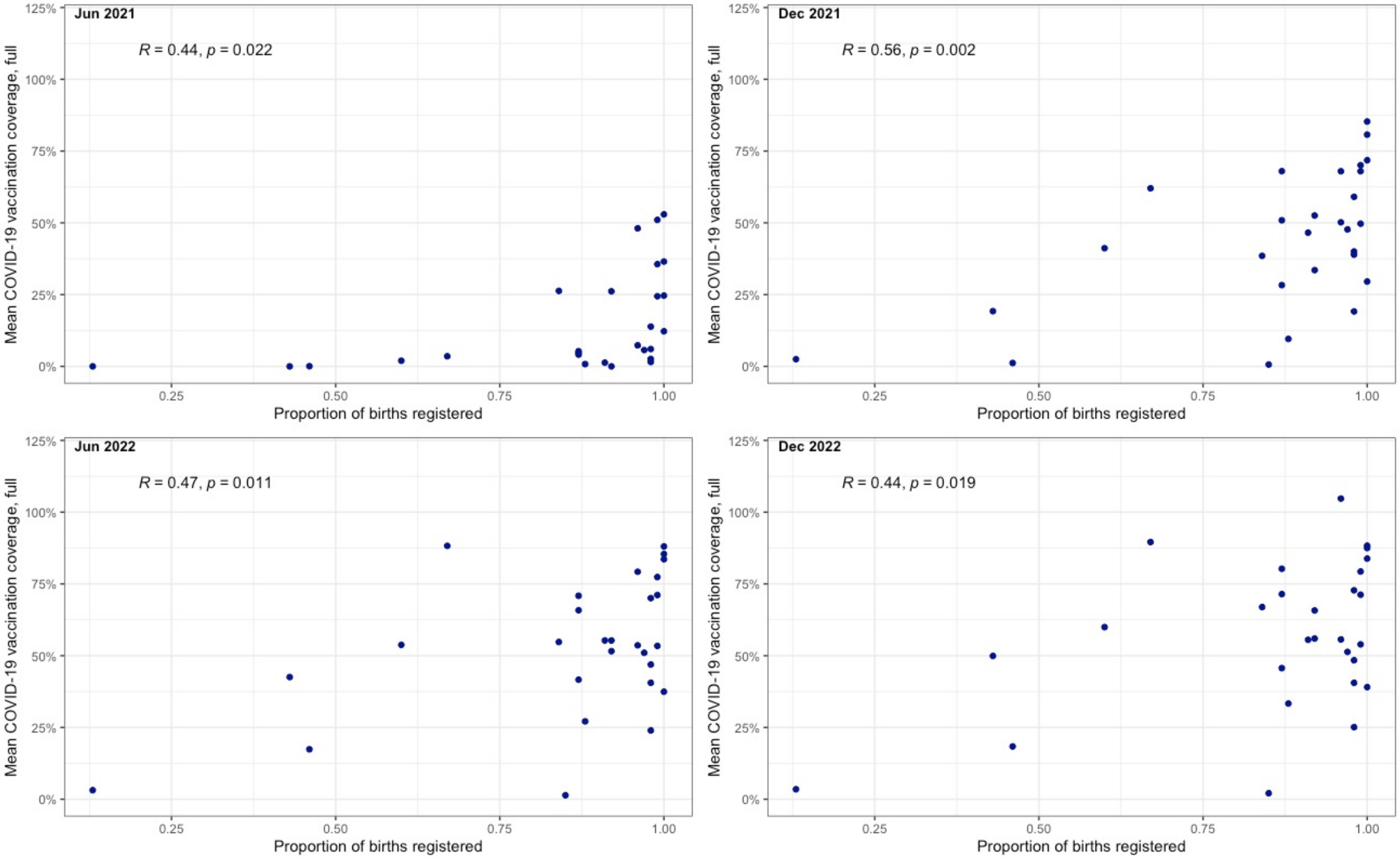

7B. Universal Health Coverage (UHC) index

Coverage of first dose of COVID-19 vaccination

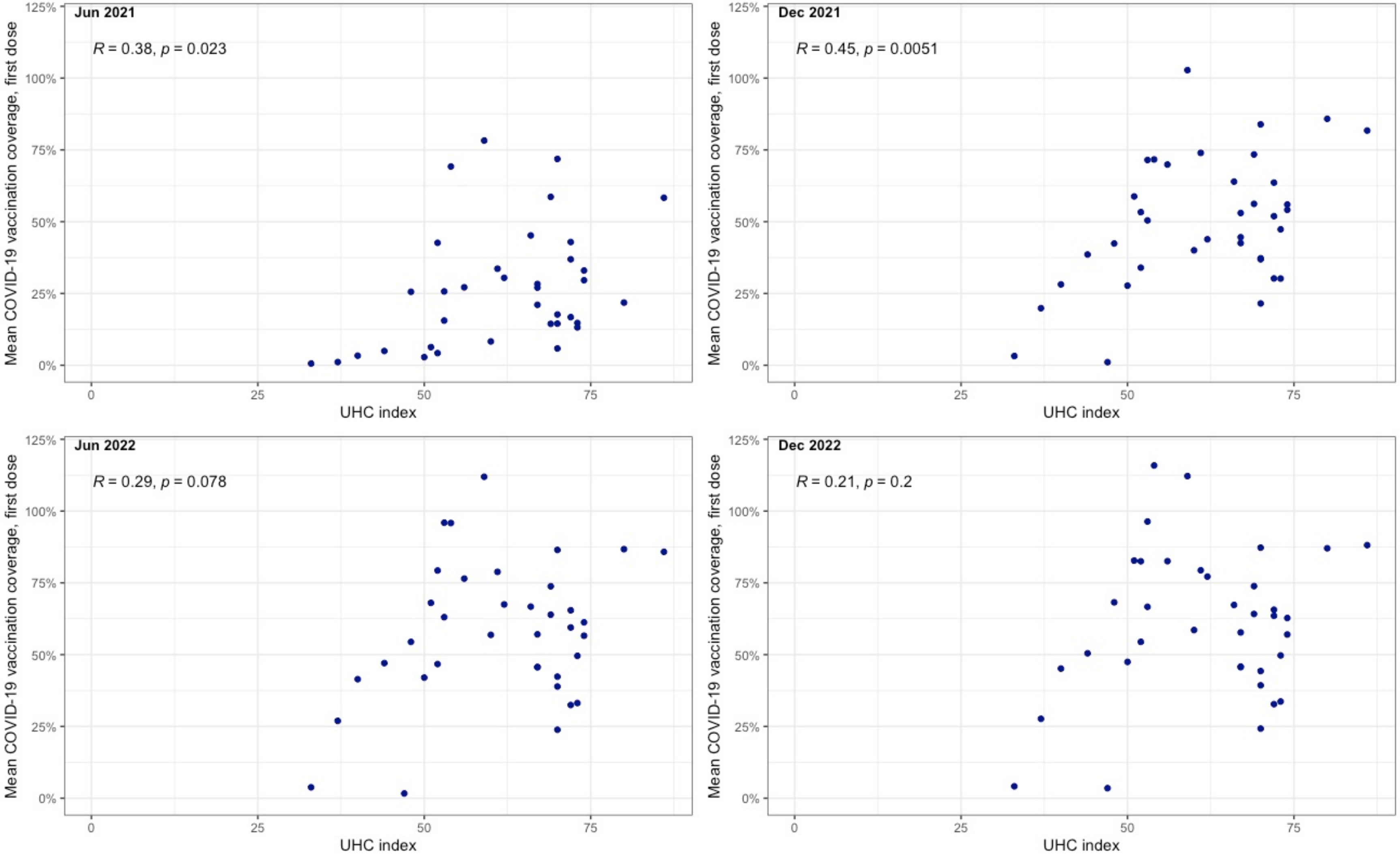

Coverage of primary series of COVID-19 vaccination

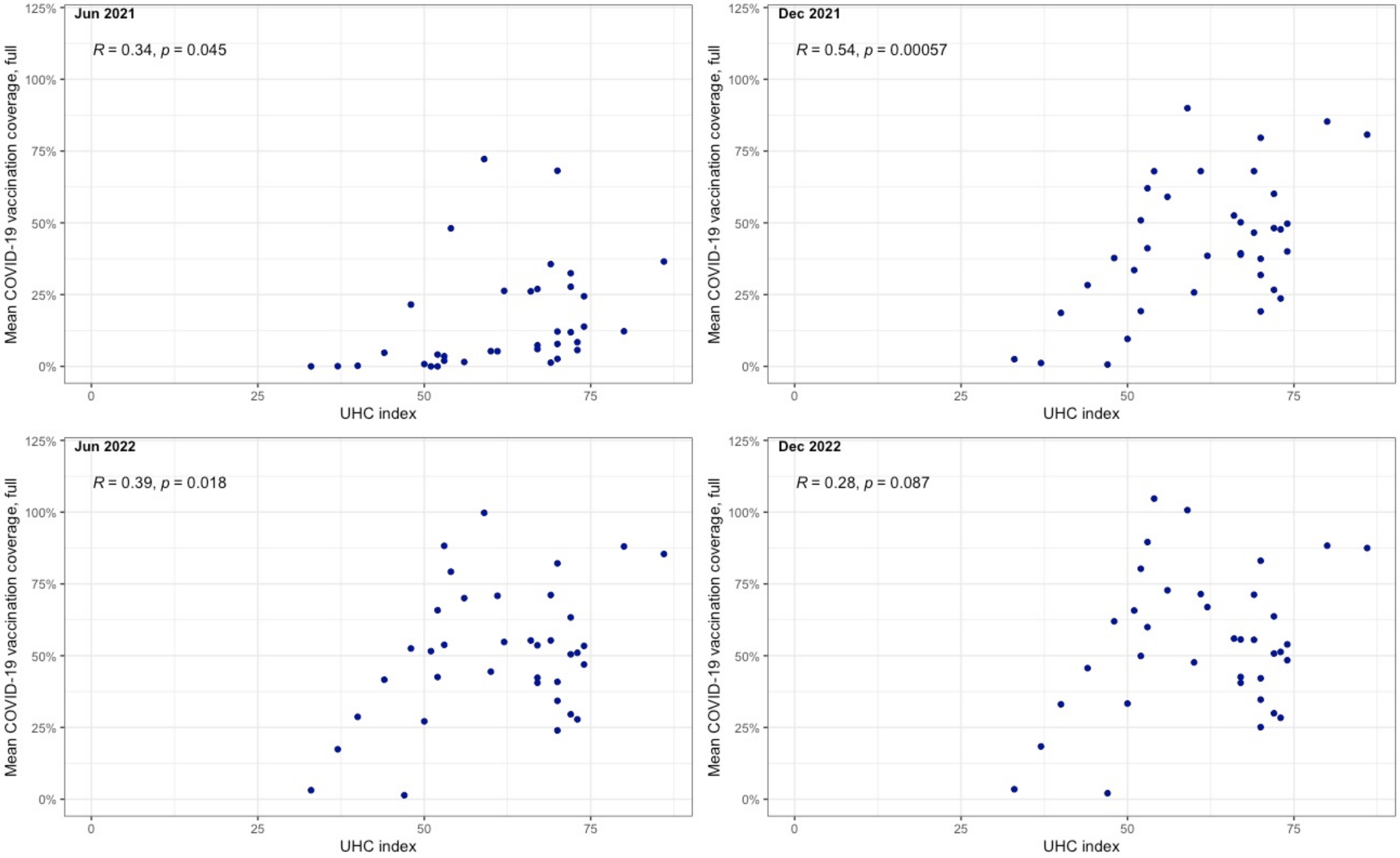

7C. Domestic government expenditure on healthcare, as a proportion of current health expenditure

Coverage of first dose of COVID-19 vaccination

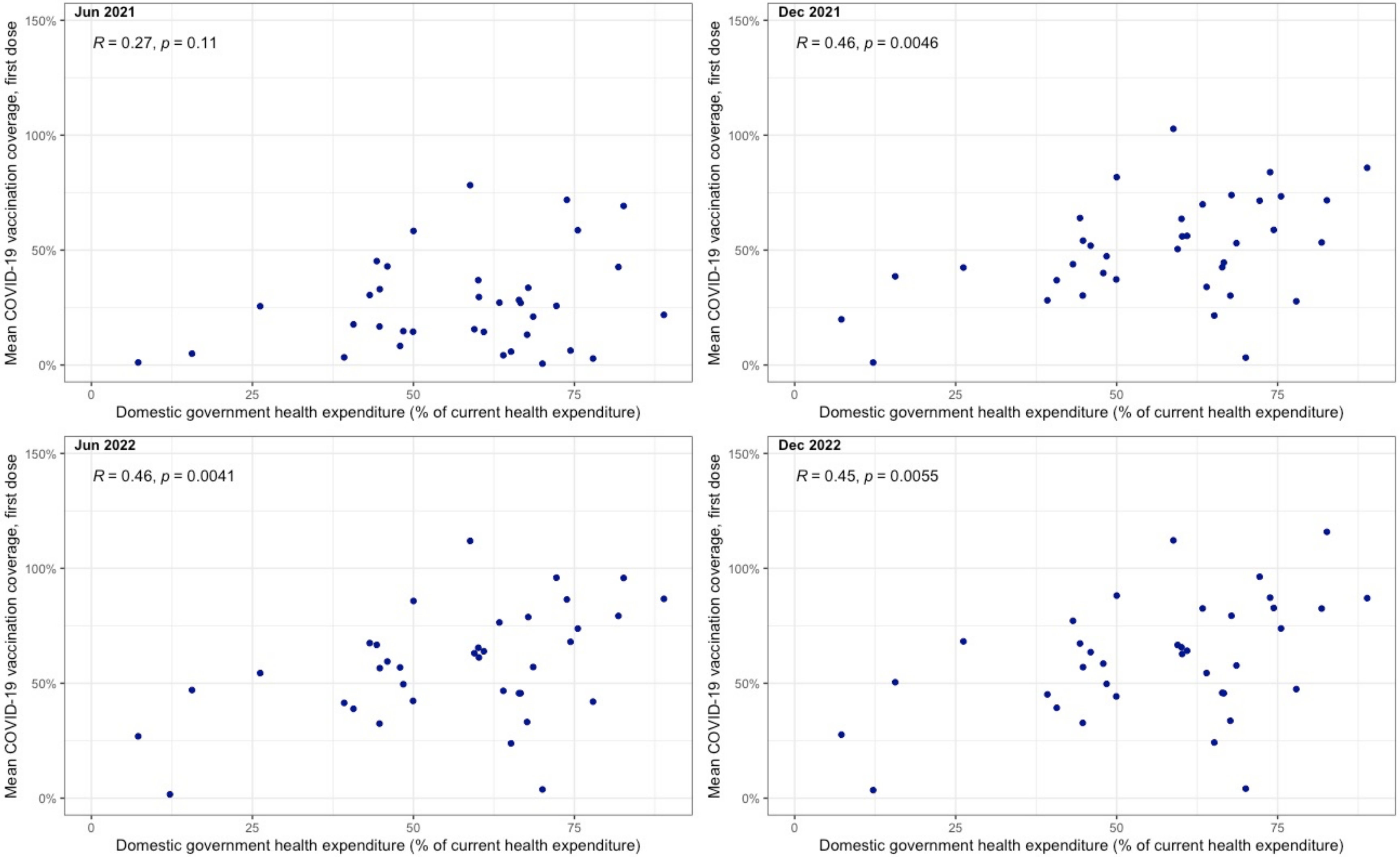

Coverage of primary series of COVID-19 vaccination

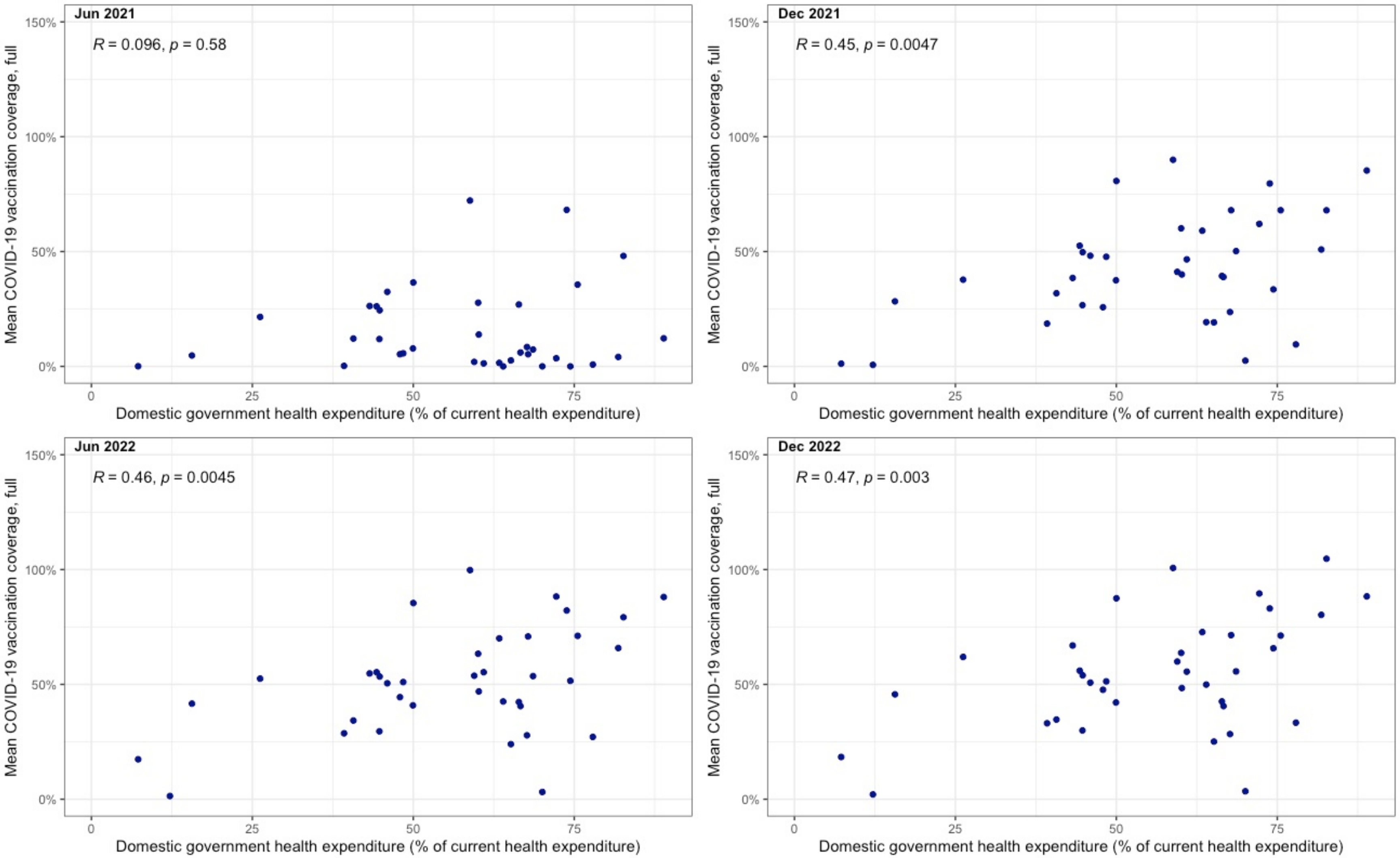

7D. Government spending on healthcare, as a proportion of total GDP

Coverage of first dose of COVID-19 vaccination

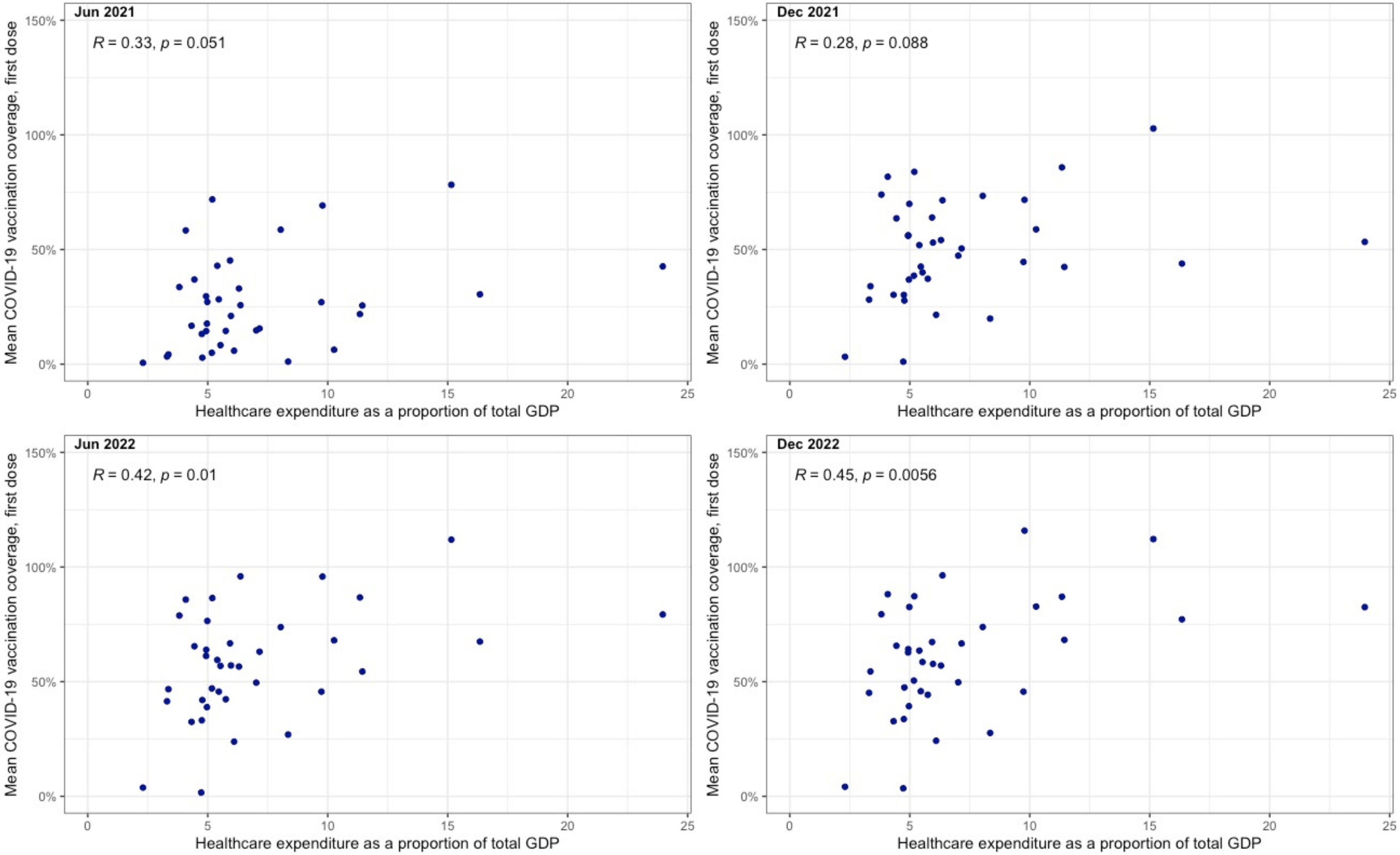

Coverage of primary series of COVID-19 vaccination

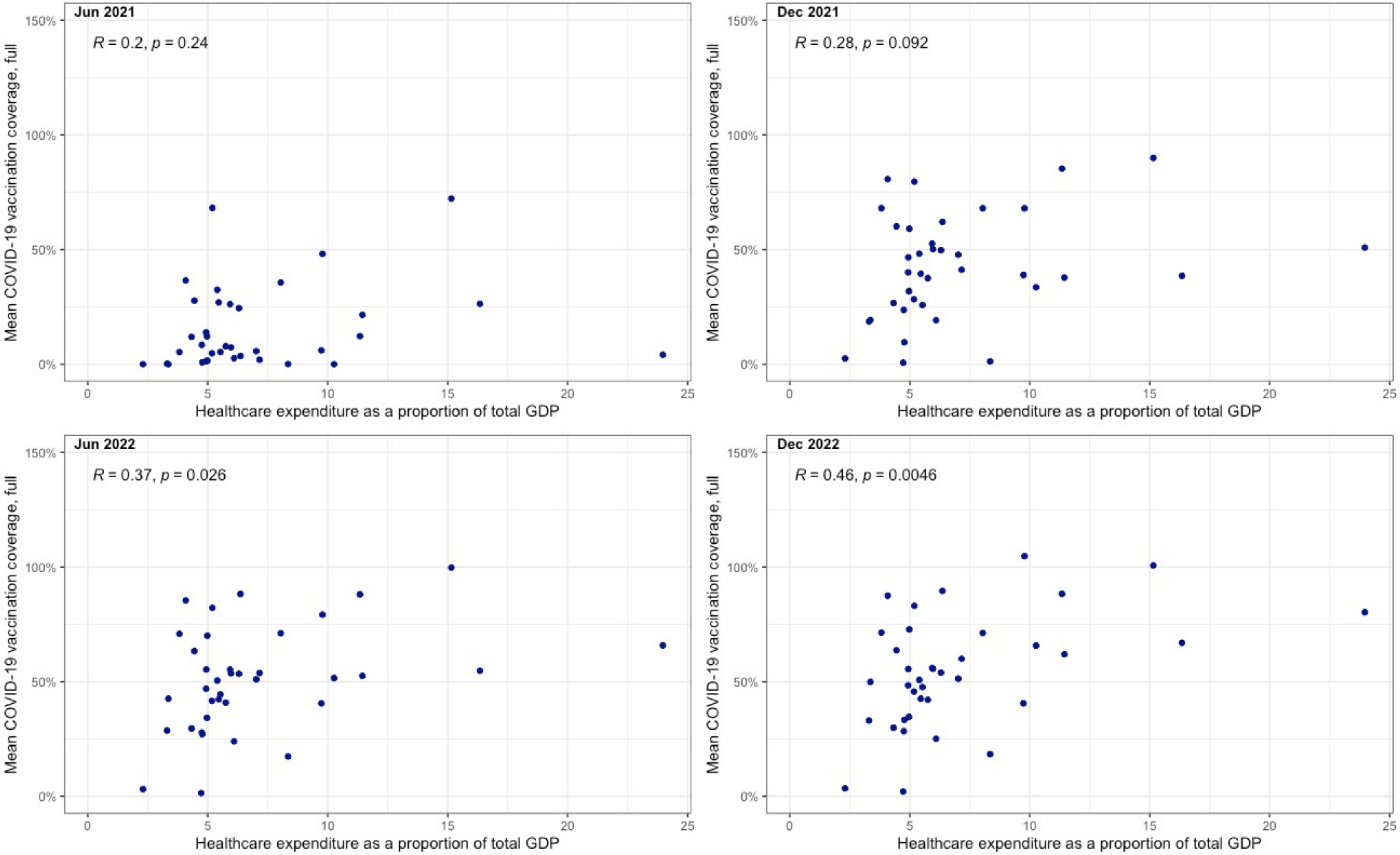

7E. Under-5 mortality rate

Coverage of first dose of COVID-19 vaccination

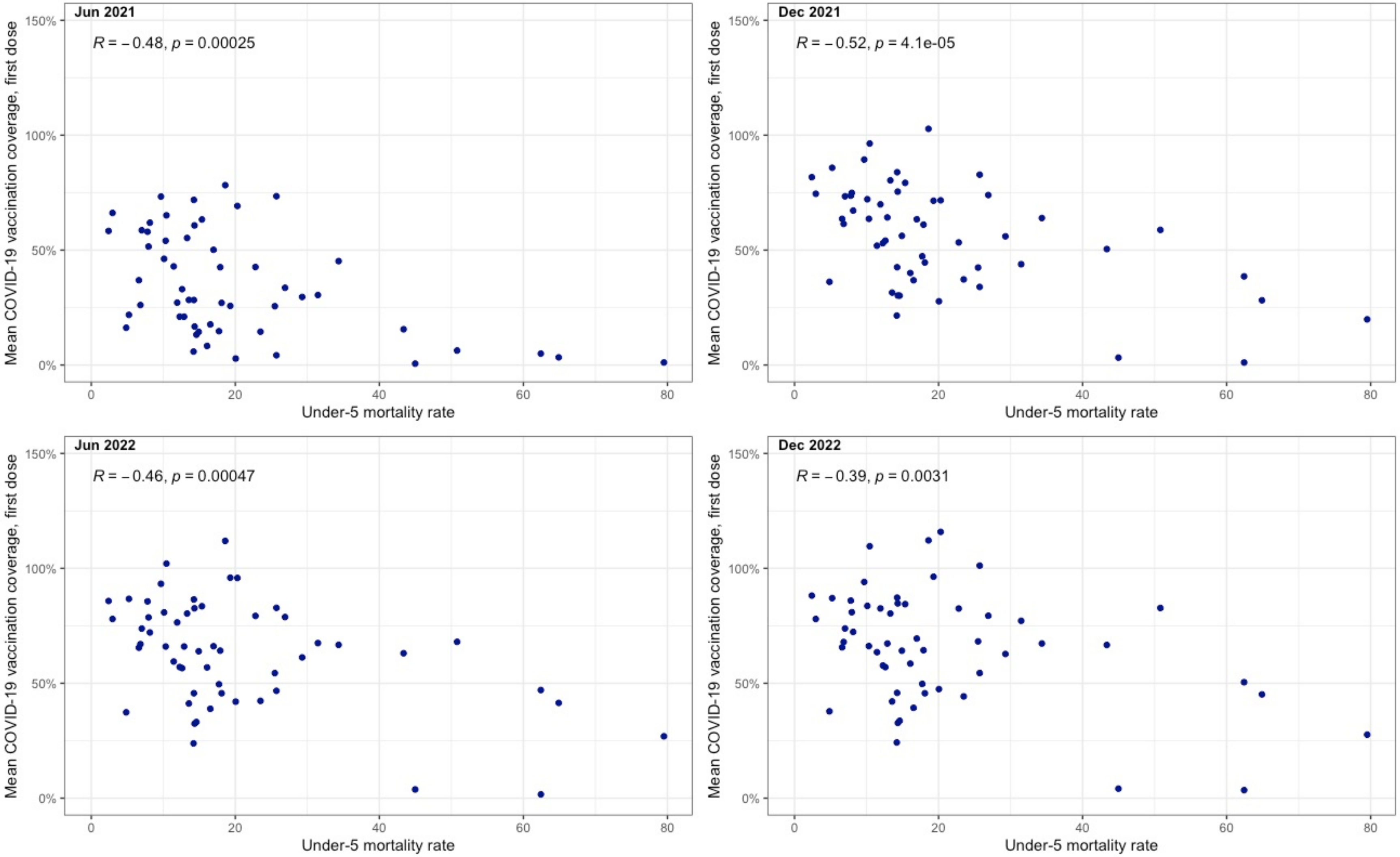

Coverage of primary series of COVID-19 vaccination

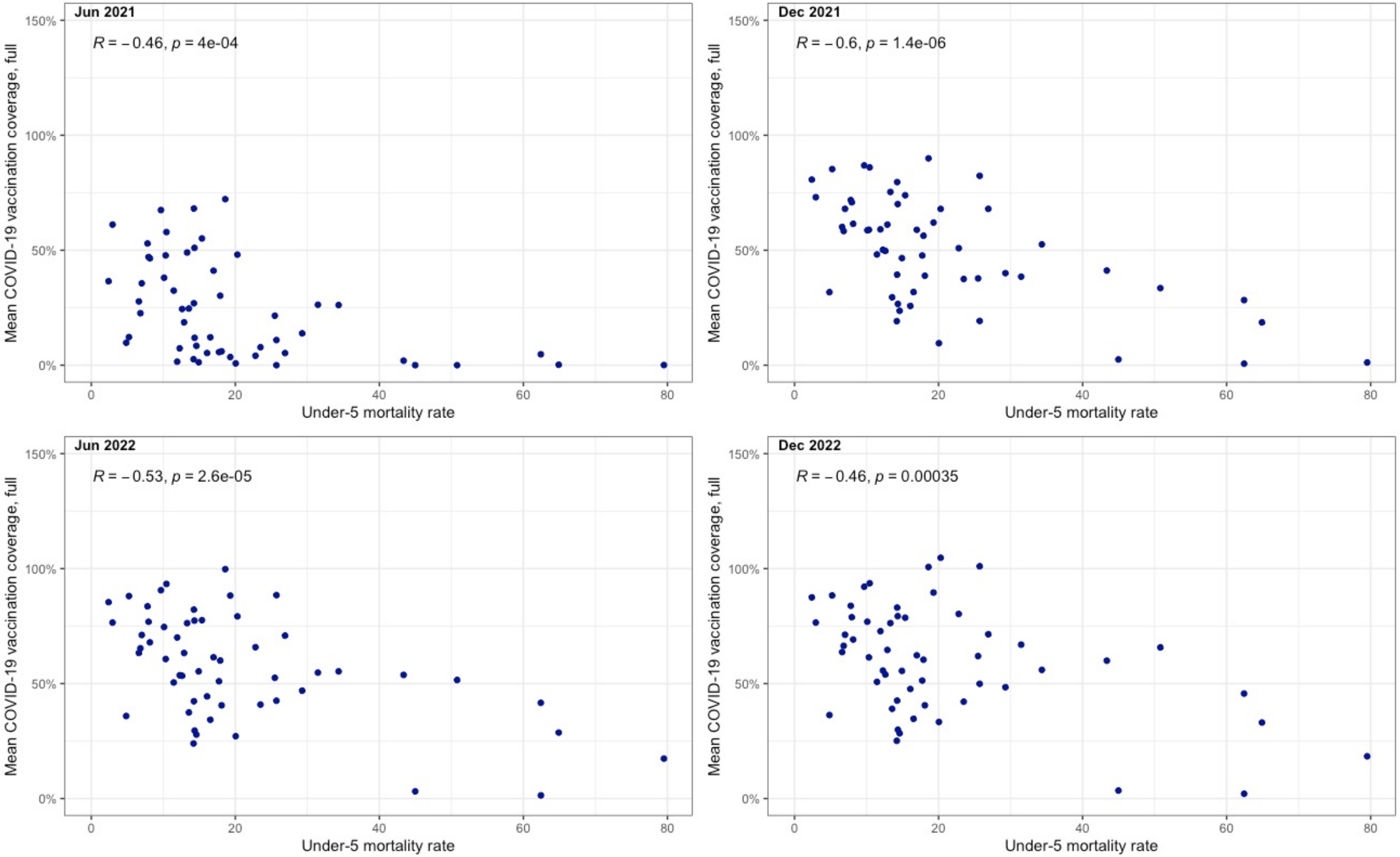

7F. Infant mortality rate

Coverage of first dose of COVID-19 vaccination

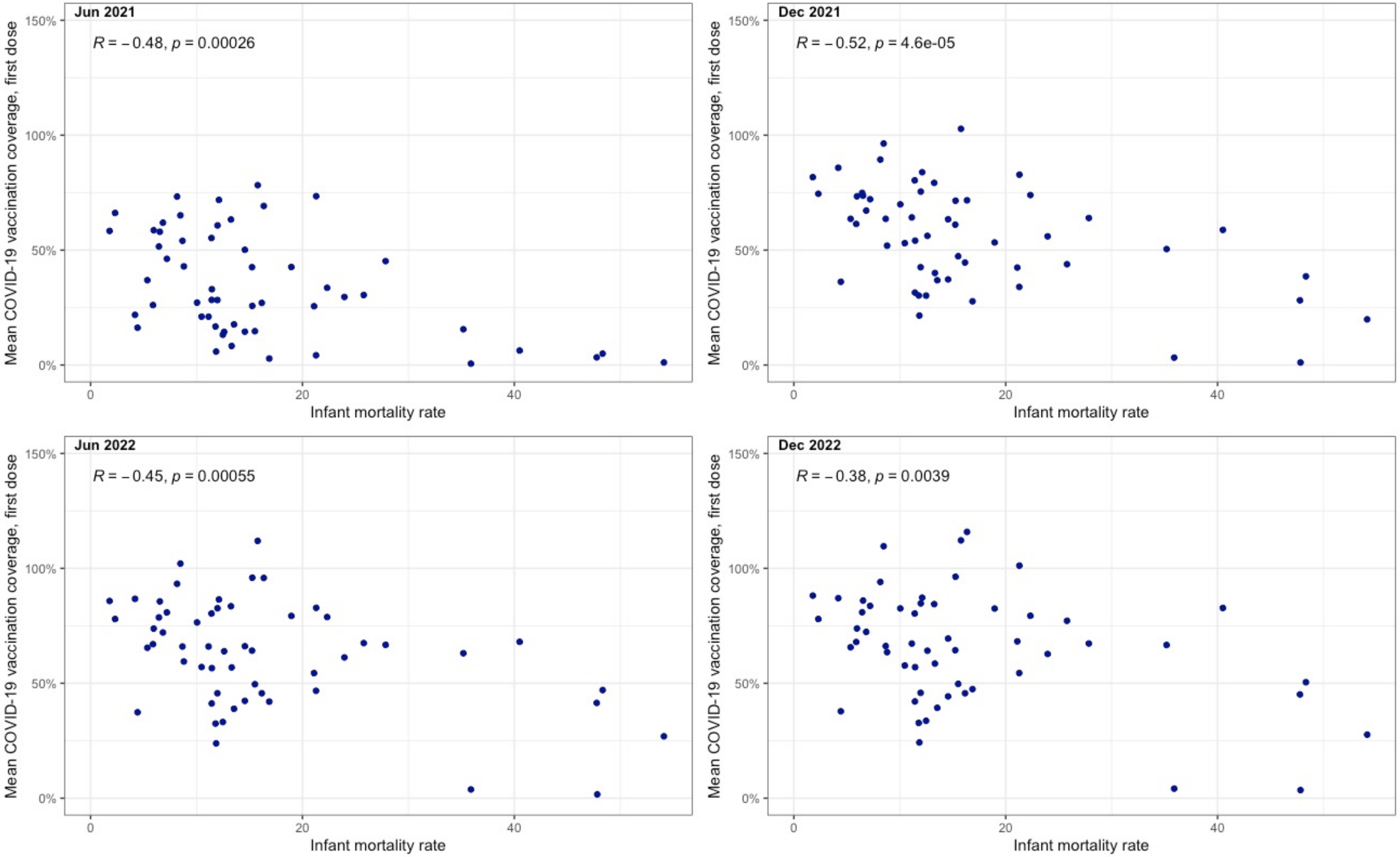

Coverage of primary series of COVID-19 vaccination

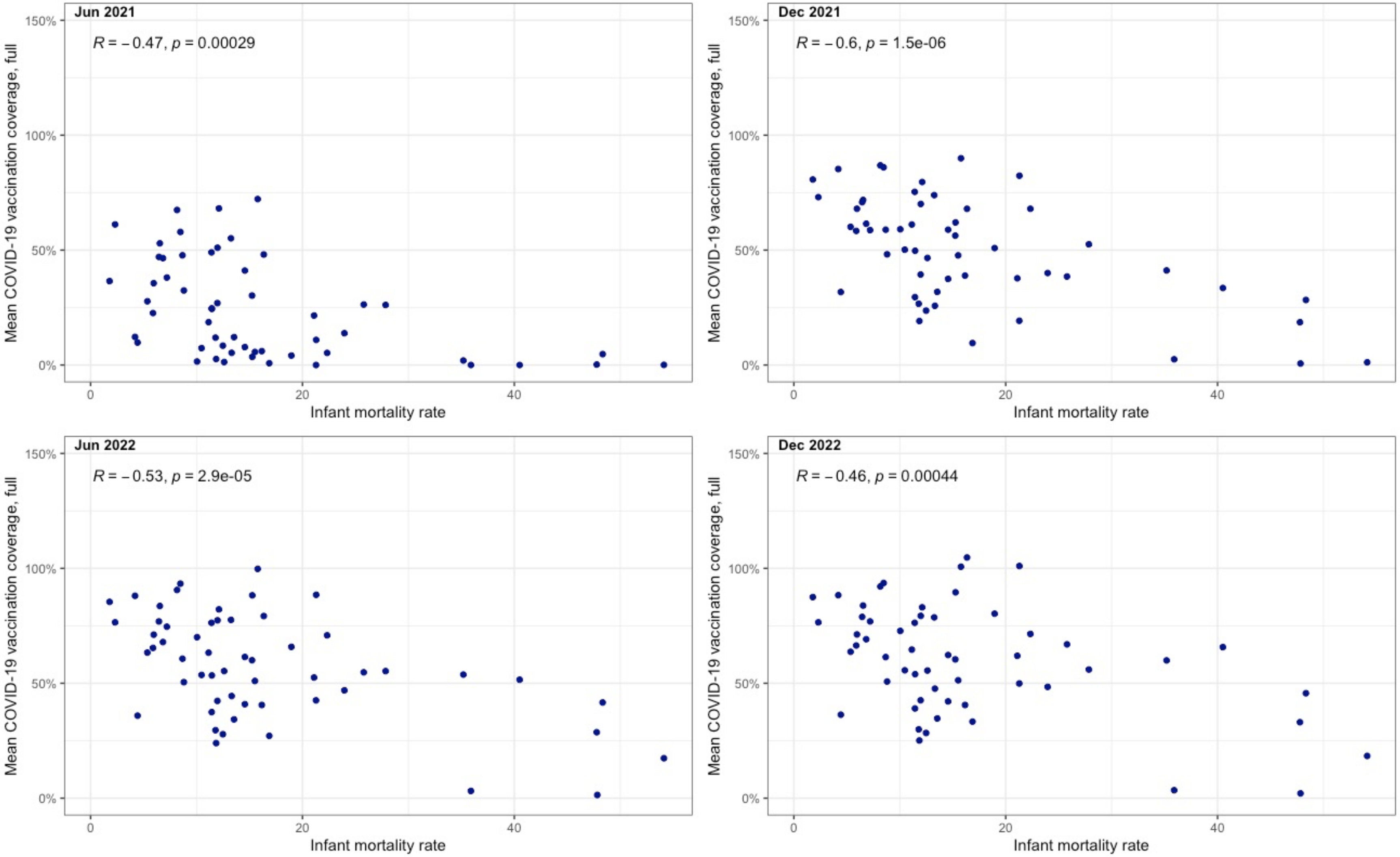

Supplement: S7 Appendix — (PDF) [file pone.0317327.s007.pdf]
