# Supplementary material for: The association between routine immunisation and COVID-19 vaccination in small Island developing states
Source: PLoS One. 2025 Jul 8;20(7):e0317327. doi: 10.1371/journal.pone.0317327 (PMC12237071; doi:10.1371/journal.pone.0317327)
Supplement: S8 Appendix — (PDF) [file pone.0317327.s008.pdf]

S8: List of countries categorised by economic factor variables

Economic factor	Criteria for categorisation	Category	N	Countries
Country income level	Defined by Gross National Income (GNI) per capita	Low	1	Guinea-Bissau
		Lower-middle	11	Cabo Verde, Comoros, Haiti, Kiribati, Micronesia (Federated States of), Papua New Guinea, Samoa, Solomon Islands, São Tomé and Príncipe, Timor-Leste, Vanuatu
		Upper-middle	16	Belize, Cuba, Dominica, Dominican Republic, Fiji, Grenada, Jamaica, Maldives, Marshall Islands, Mauritius, Palau, Saint Lucia, Saint Vincent and the Grenadines, Suriname, Tonga, Tuvalu
		High	22	American Samoa, Antigua and Barbuda, Aruba, Bahamas, Barbados, Bermuda, British Virgin Islands, Cayman Islands, Commonwealth of Northern Marianas, Curacao, French Polynesia, Guam, Guyana, Nauru, New Caledonia, Puerto Rico, Saint Kitts and Nevis, Seychelles, Singapore, Sint Maarten, Trinidad and Tobago, Turks and Caicos Islands
		Other	5	Anguilla, Cook Islands, Guadeloupe, Montserrat, Niue
Status as a least developed country	Defined based on having low GNI, an economic vulnerability index, and having low levels of human resources (based on indicators of nutrition, health, education and adult literacy)	Least developed	7	Comoros, Guinea-Bissau, Haiti, Kiribati, Solomon Islands, São Tomé and Príncipe, Tuvalu
		Developing	48	American Samoa, Anguilla, Antigua and Barbuda, Aruba, Bahamas, Barbados, Belize, Bermuda, British Virgin Islands, Cabo Verde, Cayman Islands, Commonwealth of Northern Marianas, Cook Islands, Cuba, Curacao, Dominica, Dominican Republic, Fiji, French Polynesia, Grenada, Guadeloupe, Guam, Guyana, Jamaica, Maldives, Marshall Islands, Mauritius, Micronesia (Federated States of), Montserrat, Nauru, New Caledonia, Niue, Palau, Papua New Guinea, Puerto Rico, Saint Lucia, Saint Vincent and the Grenadines, Saint Kitts and Nevis, Samoa, Seychelles, Singapore, Sint Maarten, Suriname, Timor-Leste, Tonga, Trinidad and Tobago, Turks and Caicos Islands, Vanuatu
Eligibility for Gavi funding	Based primarily on GNI per capita, with consideration of fragility and emergencies and country capacity to transition out of Gavi support	Yes	7	Comoros, Guinea-Bissau, Haiti, Mauritius, Papua New Guinea, Solomon Islands, São Tomé and Príncipe
		No	32	American Samoa, Antigua and Barbuda, Bahamas, Barbados, Belize, Bermuda, Cabo Verde, Commonwealth of Northern Marianas, Cuba, Dominica, Dominican Republic, Fiji, Grenada, Guam, Guyana, Jamaica, Kiribati, Maldives, Marshall Islands, Micronesia (Federated States of), Puerto Rico, Saint Lucia, Saint Vincent and the Grenadines, Samoa, Seychelles, Singapore, Suriname, Timor-Leste, Tonga, Trinidad and Tobago, Vanuatu
		N/A (non-WHO member states)	16	Anguilla, Aruba, British Virgin Islands, Cayman Islands, Cook Islands, Curacao, French Polynesia, Guadeloupe, Montserrat, Nauru, New Caledonia, Niue, Palau, Saint Kitts and Nevis, Sint Maarten, Turks and Caicos Islands, Tuvalu
COVAX status	Based primarily on the country's income classification with all low and lower-middle income countries being eligible, and	Self-financing	12	Antigua and Barbuda, Bahamas, Barbados, Belize, Dominican Republic, Jamaica, Nauru, Palau, Saint Kitts and Nevis, Singapore, Suriname, Trinidad and Tobago
		AMC	23	Cabo Verde, Comoros, Dominica, Fiji, Grenada, Guinea-Bissau, Guyana, Haiti, Kiribati, Maldives, Marshall Islands, Mauritius, Micronesia (Federated States of), Papua New

Economic factor	Criteria for categorisation	Category	N	Countries
	some upper-middle income countries eligible based on criteria related to economic vulnerability			Guinea, Saint Lucia, Saint Vincent and the Grenadines, Samoa, Solomon Islands, São Tomé and Príncipe, Timor-Leste, Tonga, Tuvalu, Vanuatu
		No	4	Cook Islands, Cuba, Niue, Seychelles
		N/A (non-WHO member states)	16	American Samoa, Anguilla, Aruba, Bermuda, British Virgin Islands, Cayman Islands, Commonwealth of Northern Marianas, Curacao, French Polynesia, Guadeloupe, Guam, Montserrat, New Caledonia, Puerto Rico, Sint Maarten, Turks and Caicos Islands

AMC = Advanced Market Commitment; GNI = Gross National Income; WHO = World Health Organization
