# Supplementary material for: The association between routine immunisation and COVID-19 vaccination in small Island developing states
Source: PLoS One. 2025 Jul 8;20(7):e0317327. doi: 10.1371/journal.pone.0317327 (PMC12237071; doi:10.1371/journal.pone.0317327)

S9: Scatterplots of COVID-19 vaccination coverage and demographic factors included in the study

9A. Proportion of population living in rural areas

Coverage of first dose of COVID-19 vaccination

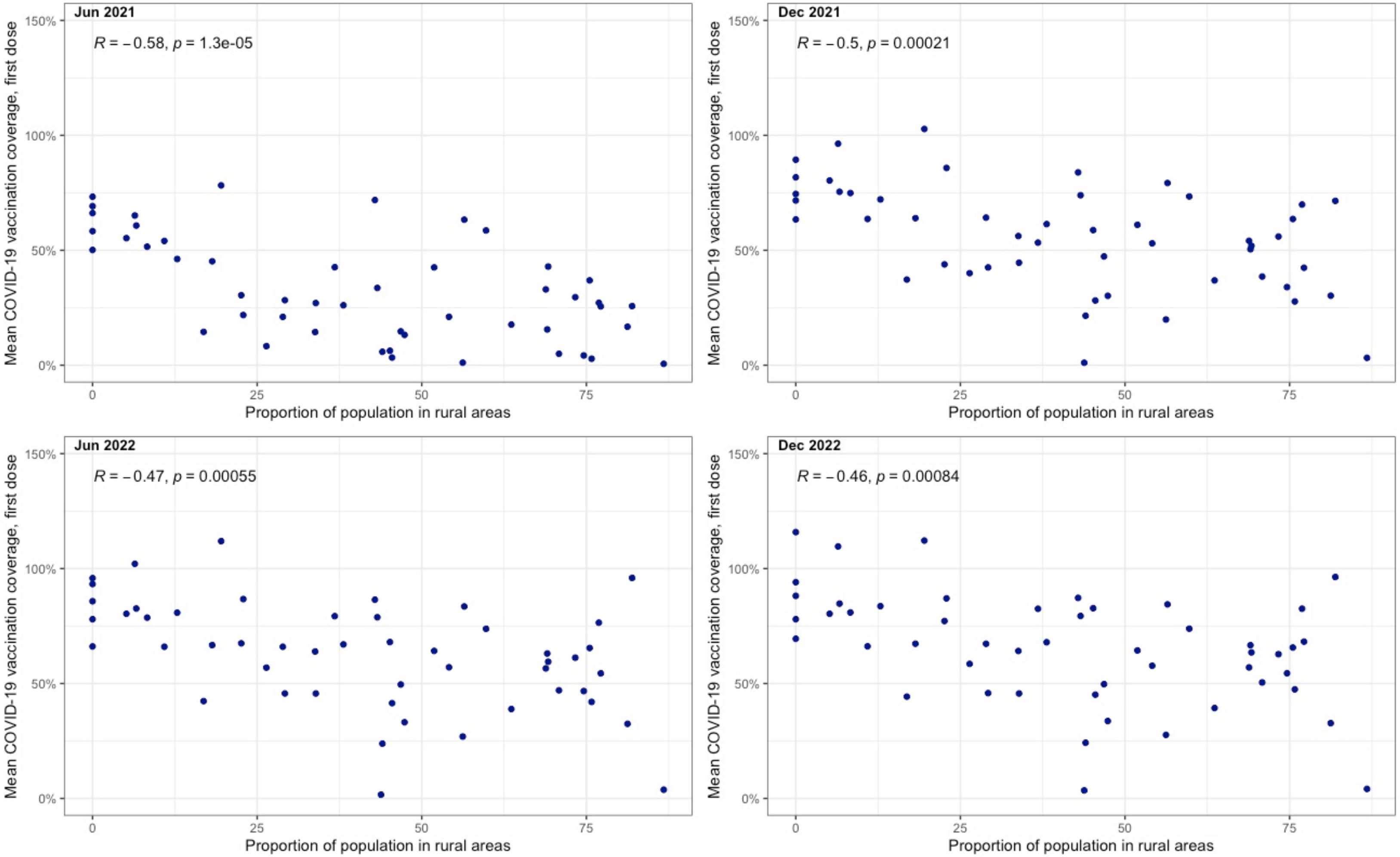

Coverage of primary series of COVID-19 vaccination

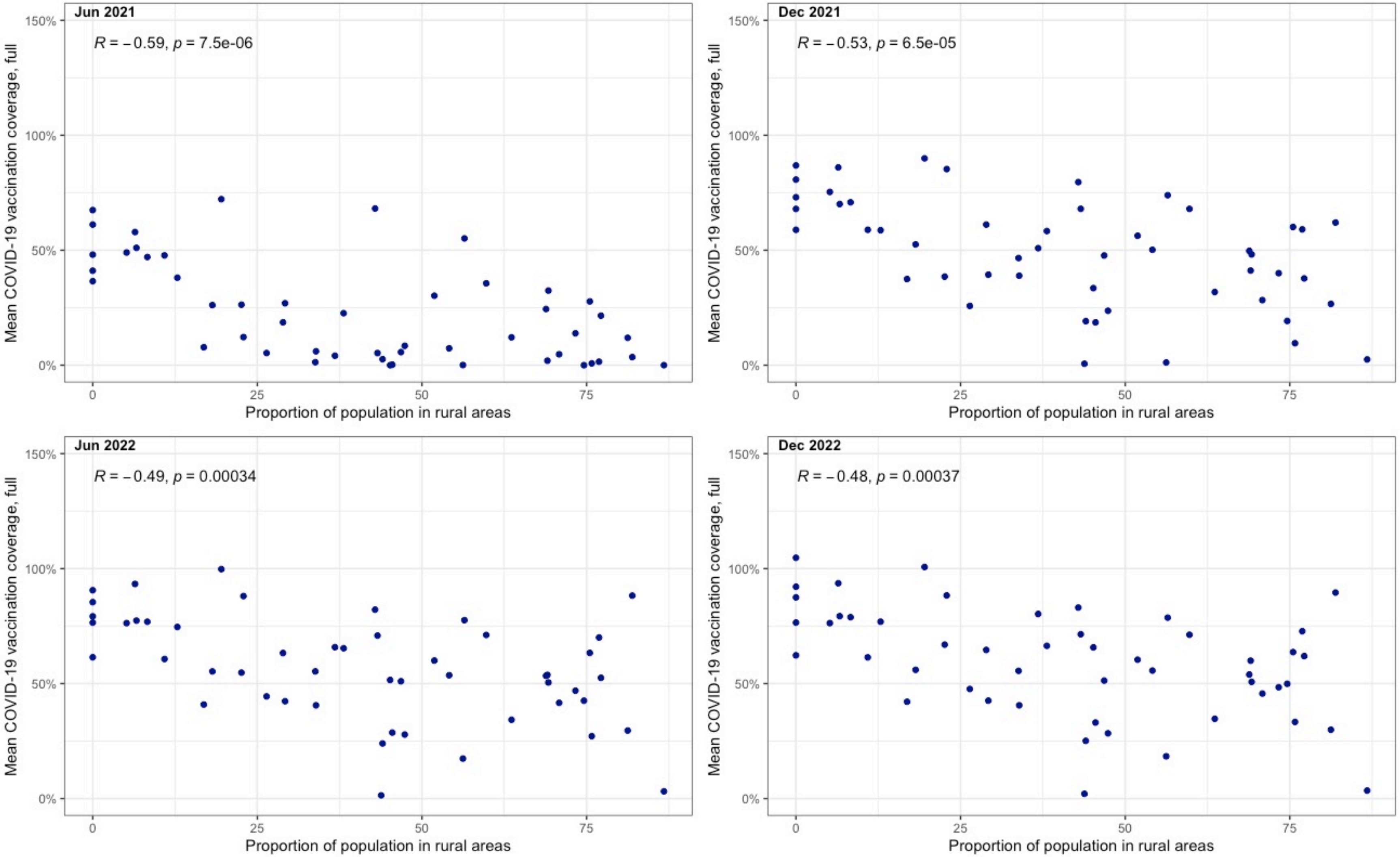

9B. Population density

Coverage of first dose of COVID-19 vaccination

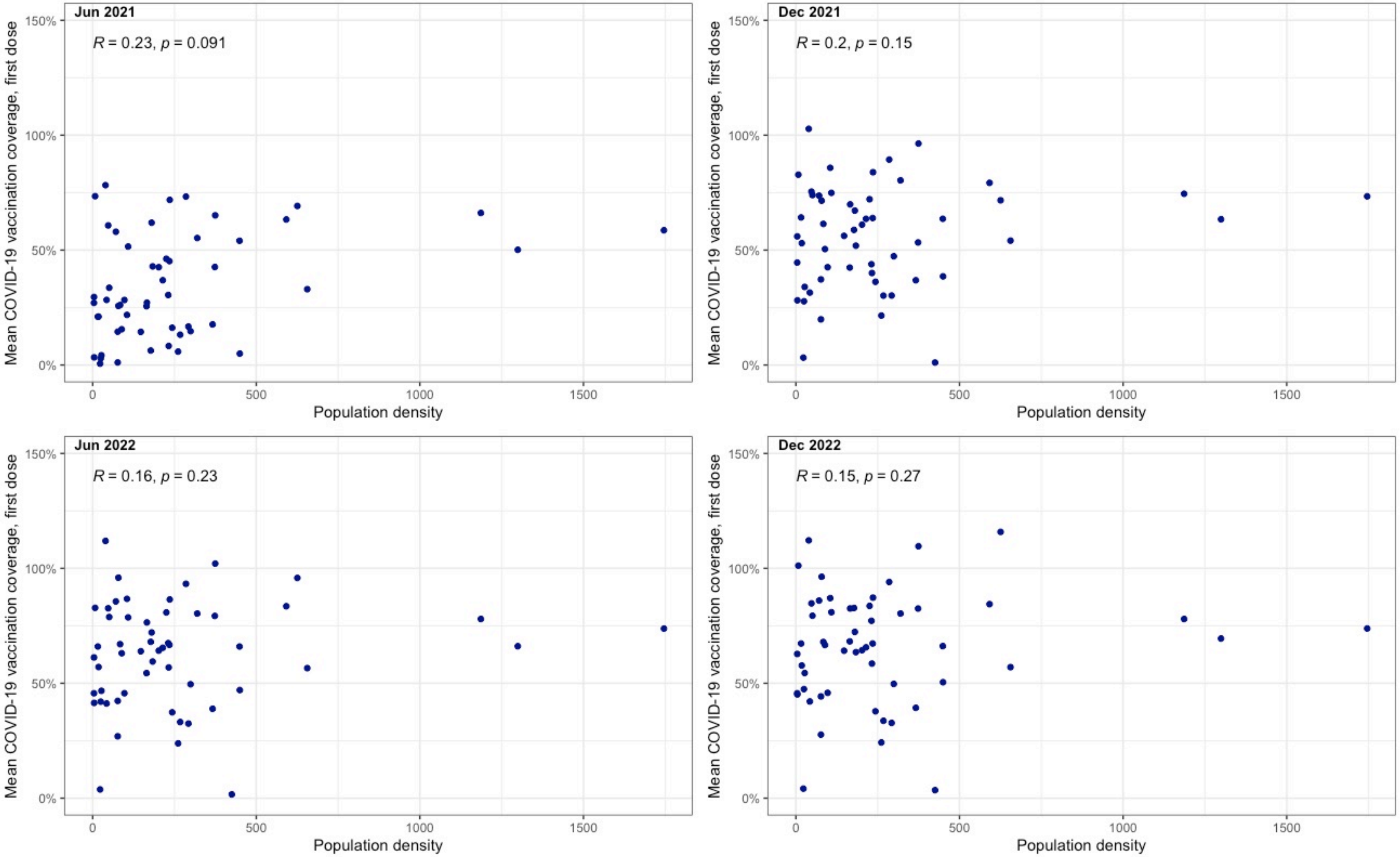

Coverage of primary series of COVID-19 vaccination

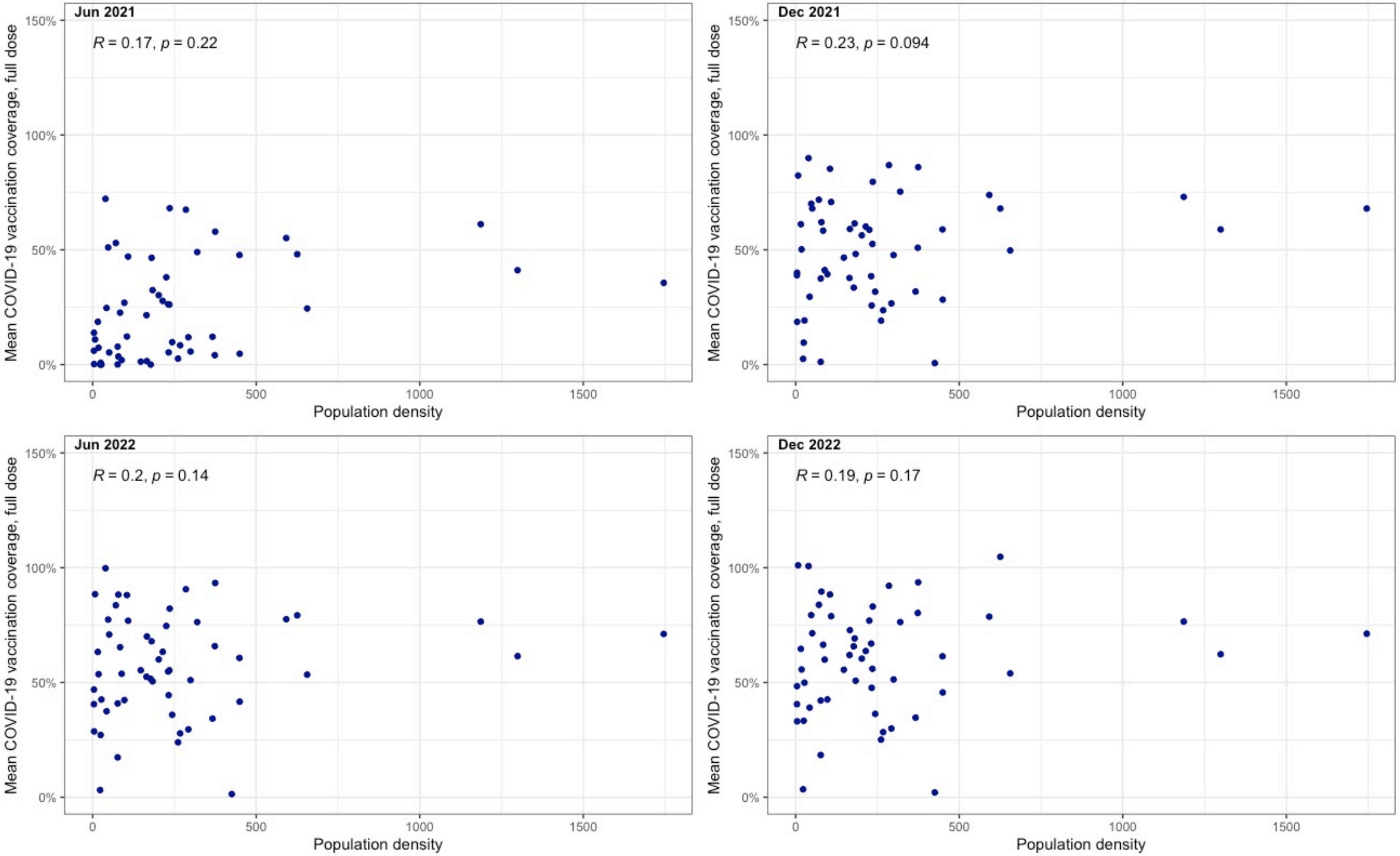

*9C. Population size**

Coverage of first dose of COVID-19 vaccination

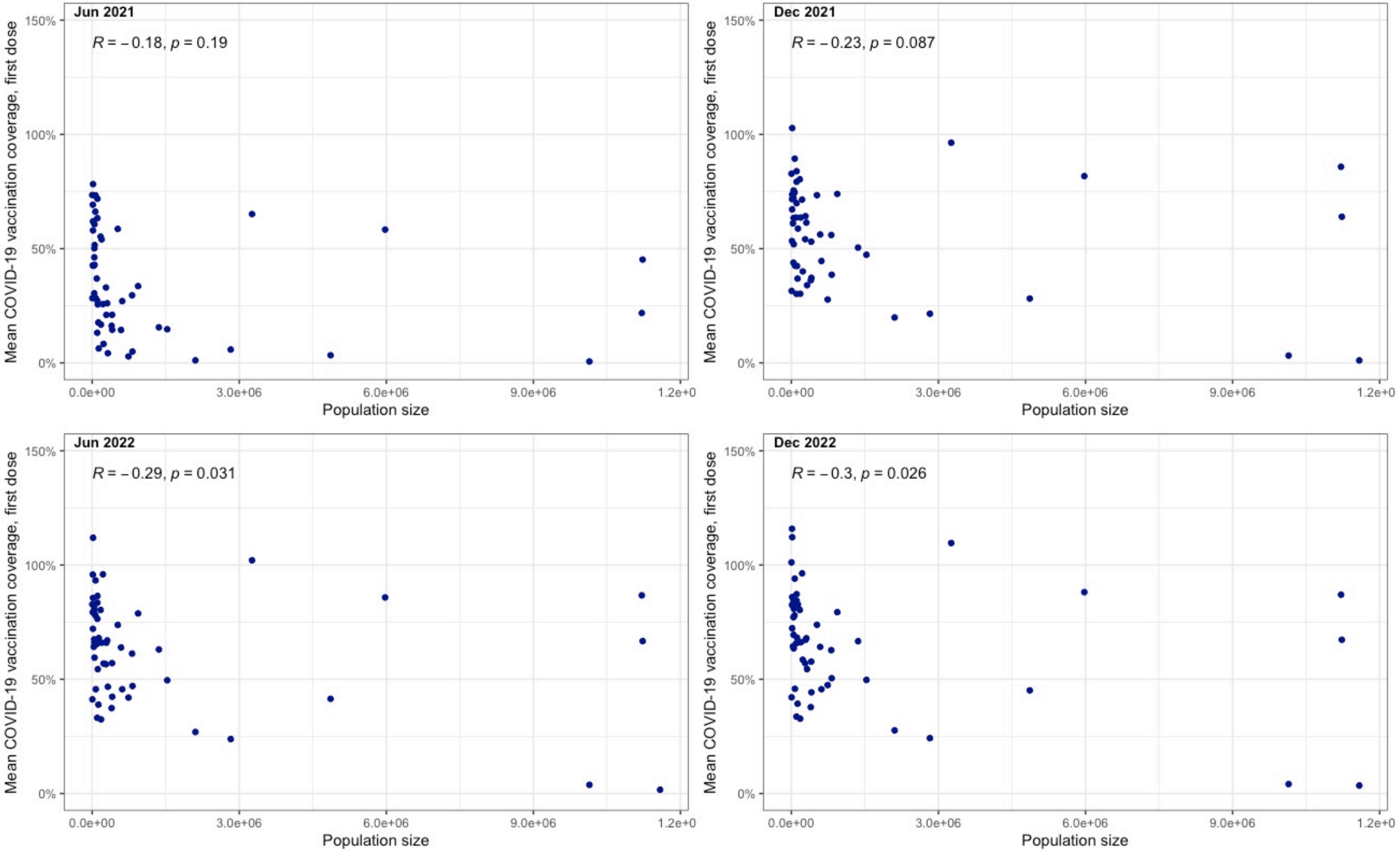

Coverage of primary series of COVID-19 vaccination

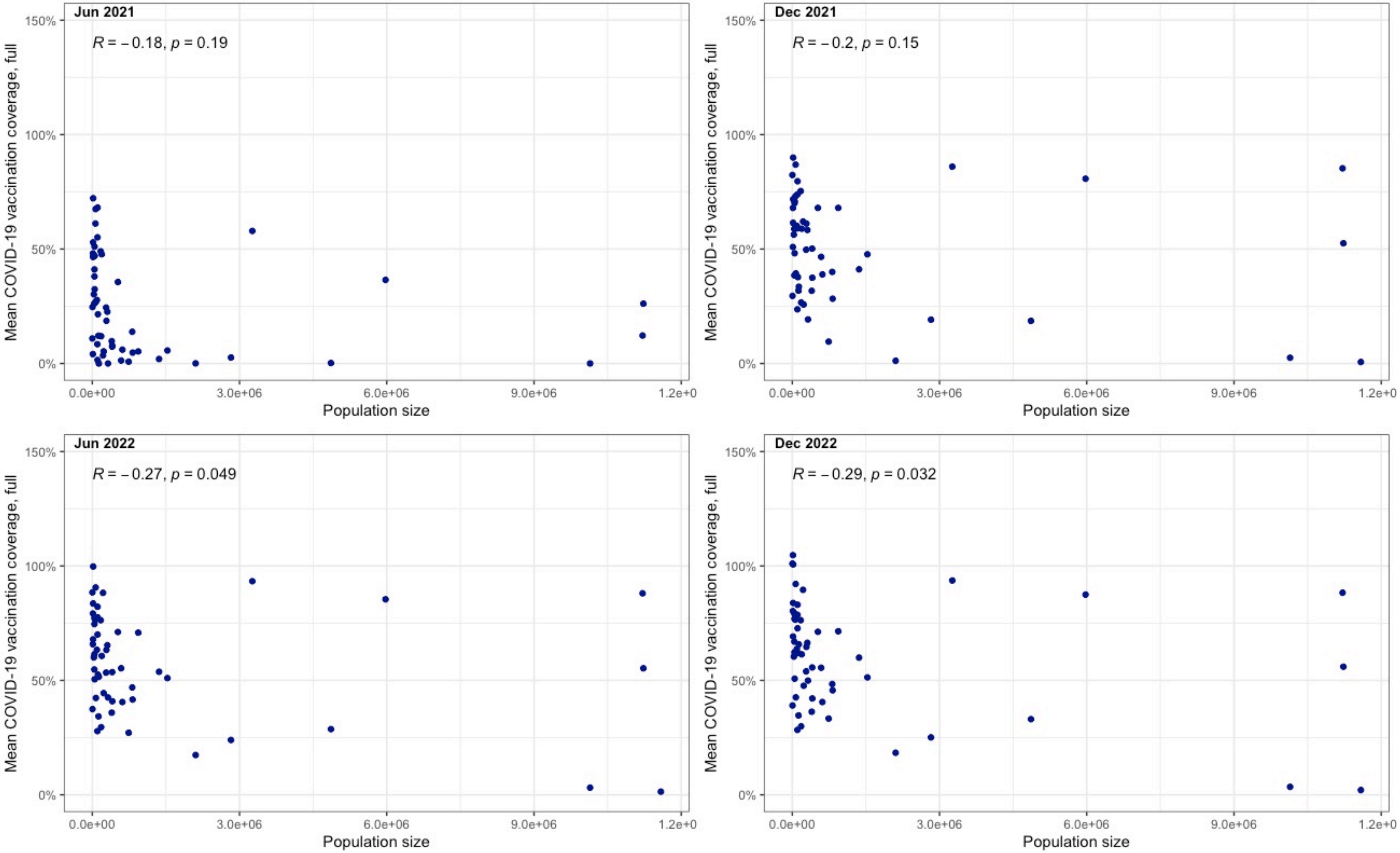

* One country was an outlier and excluded

9D. Gender parity index

Coverage of first dose of COVID-19 vaccination

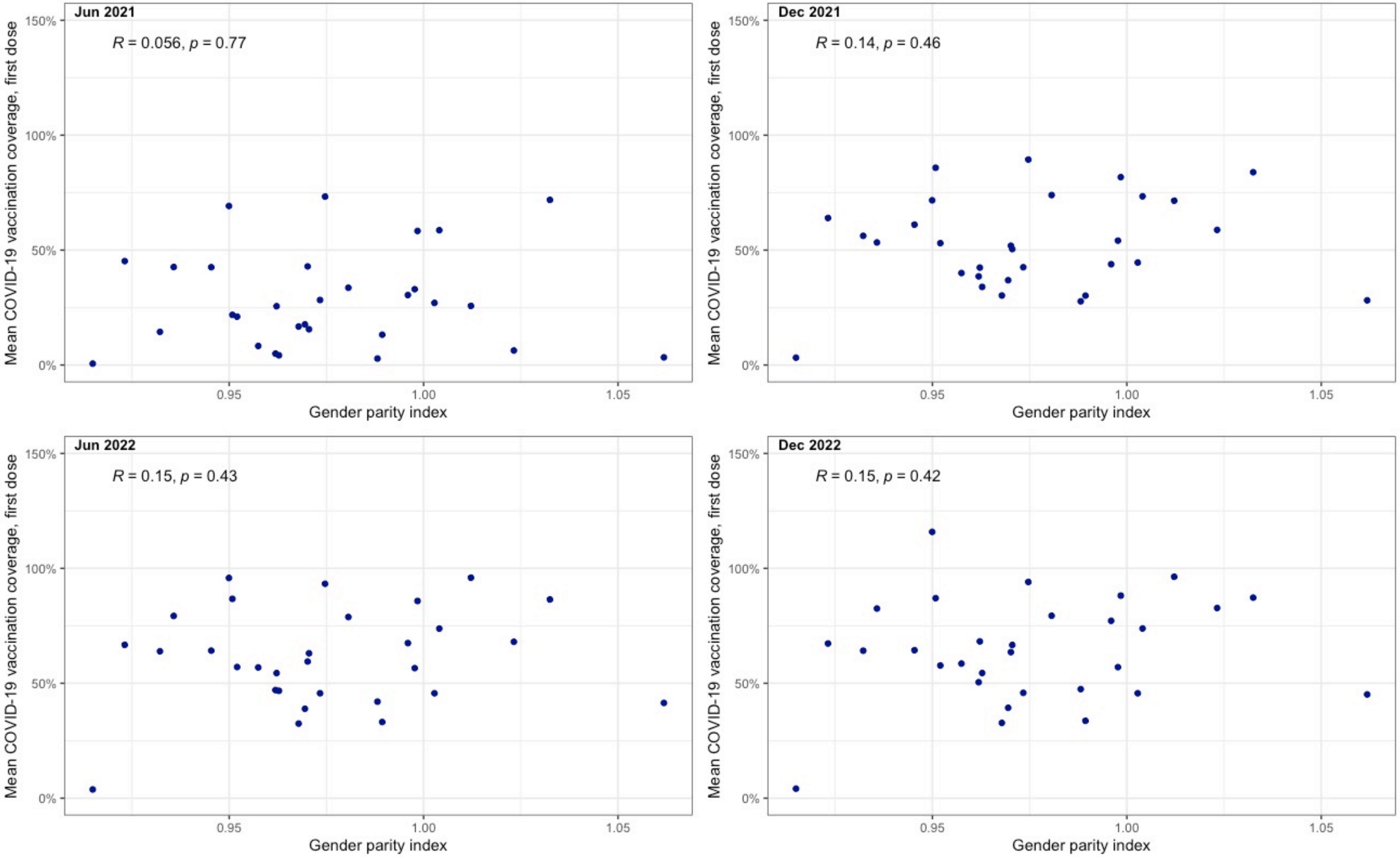

Coverage of primary series of COVID-19 vaccination

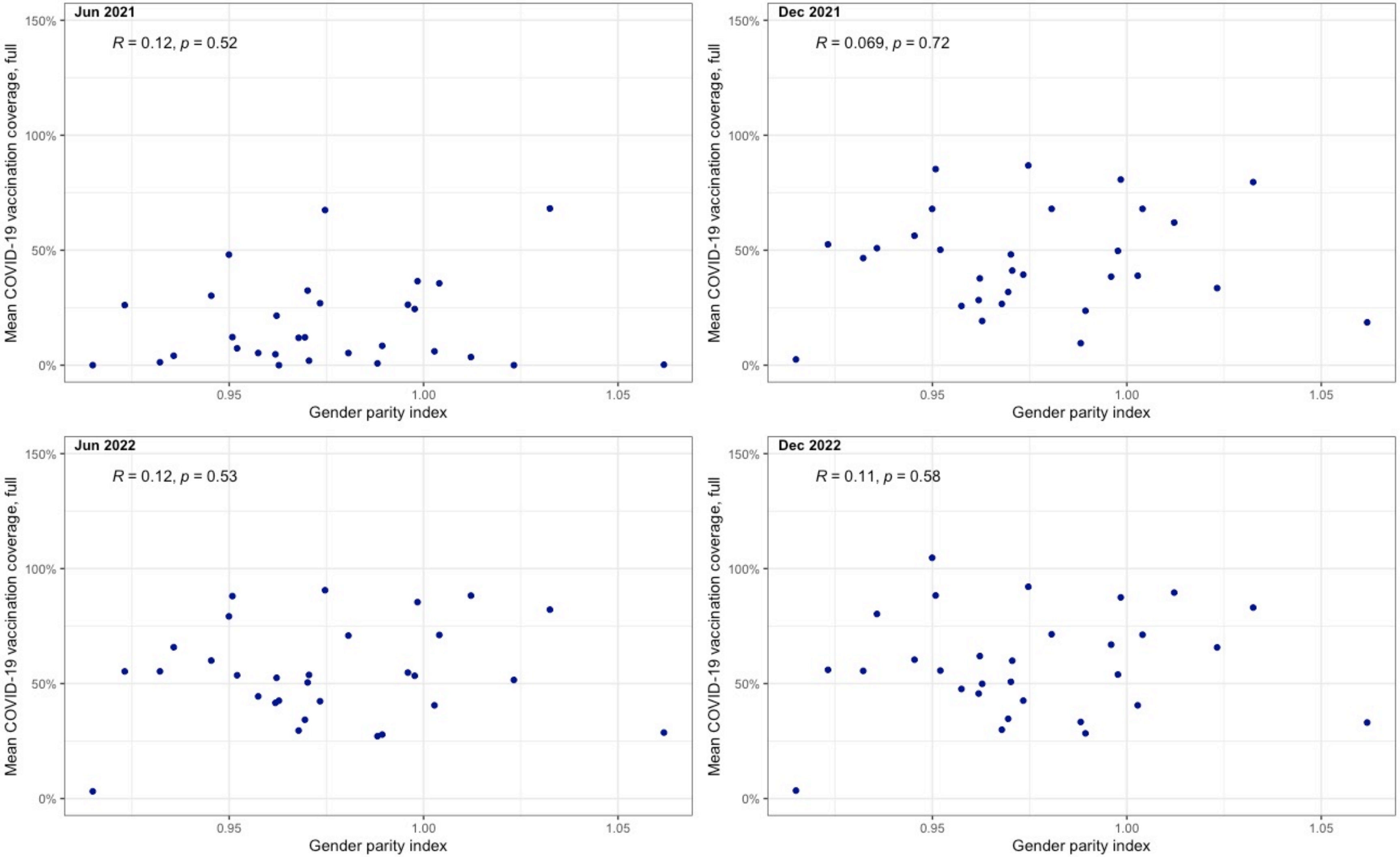

Supplement: S9 Appendix — (PDF) [file pone.0317327.s009.pdf]
