# Supplementary material for: The association between routine immunisation and COVID-19 vaccination in small Island developing states
Source: PLoS One. 2025 Jul 8;20(7):e0317327. doi: 10.1371/journal.pone.0317327 (PMC12237071; doi:10.1371/journal.pone.0317327)
Supplement: Attachment — Submitted filename: PLOS_routine immunisation and COVID-19 vaccination_26012025.docx [file pone.0317327.s010.docx]

The manuscript with the title *"The association between routine immunisation and COVID-19 vaccination in small island developing states"* aimed to understanding the link between routine immunisation (RI) performance and vaccination during an epidemic. The study used multiple data sources (i.e., WHO/UNICEF Joint Reporting Form, World Bank Indicators) to enhance the robustness of the findings. In addition, the study collected information on health system factors, economic indicators, and demographic characteristics, allows for a multifaceted analysis. Below are the comments need to be addressed before consideration for publication.

1. The study depends primarily on Spearman correlations, which cannot prove causation. The analysis limits the capacity to draw firm conclusions regarding causative pathways. The study lacks extensive statistical modelling (e.g., regression analysis).
2. Inconsistent data from non-WHO member states might limits the findings' applicability to all SIDS.
3. The use of publicly available datasets may result in biases or inaccuracies due to different reporting standards and operation standards.
4. The study acknowledges the impact of health system resilience but does not delve into other important elements such community trust, vaccine hesitancy, and political pressures.
5. Another limitation of the current is that the study does not account for potential lag effects between routine immunization performance and COVID-19 vaccination uptake.
6. The study mentions supply issues. There are other important factors which are not considered in the current study including how geopolitical factors (e.g., vaccine diplomacy, reliance on external donors) impacted vaccine access in SIDS.
7. The study depends on countries with good reporting system for Routine Immunisation.
8. The focus of this study is on aggregate country-level statistics does not consider within-country differences, which may be considerable in SIDS.
9. The study shall provide more specific recommendation based on the findings, particularly in resource-limited areas.
