# Supplementary material for: The association between routine immunisation and COVID-19 vaccination in small Island developing states
Source: PLoS One. 2025 Jul 8;20(7):e0317327. doi: 10.1371/journal.pone.0317327 (PMC12237071; doi:10.1371/journal.pone.0317327)
Supplement: Attachment — Submitted filename: Author responses to comments_final_2025.03.05.docx [file pone.0317327.s012.docx]

**The association between routine immunisation and COVID-19 vaccination in small island developing states**

**Response to reviewers’ comments**

We thank the reviewers for the comments on our paper and for the opportunity to address them. We have clarified certain aspects of our paper and included additional details in the manuscript in response. Below, we provide responses to each of the reviewers’ comments and line number references where we have made changes, or point to where existing text in the manuscript addresses comments.

**Responses to reviewer 1**

*Comment 1: Tables 1 and 2 provide independent variables and characteristics of the study sample.
Table 3: Spearman correlations between COVID-19 vaccination coverage and 5-year (2015–2019) mean annual coverage of routine Immunisations.
What is the point of studying the correlation between COVID-19 vaccination coverage and 5-year (2015–2019) mean annual coverage of routine Immunisations?*

**Response**: We analysed the correlation between countries’ 5-year mean routine vaccination coverage and COVID-19 vaccine coverage to answer our research question of whether there is a relationship between a robust childhood immunisation system and having higher coverage of vaccination delivered in response to an infectious disease outbreak. We have clarified why we have used correlation statistics in the text added to the methods section.

**Changes to manuscript**: Methods: data analyses section, page 15, line 259–260, text added:

We calculated correlation coefficients where independent variables were continuous, as they allow an investigation into the strength and direction of an association between variables. *(underlined text is additional)*

*Comment 2: Table 4: Spearman correlations between COVID-19 vaccination coverage and density of health resources (workforce and hospital beds).
I do not see any critical points from the data provided in Table 4.*

Response: We have expanded our discussion to more explicitly examine the results presented in Table 4. In our analysis, the correlation between COVID-19 vaccination coverage and physician density was the strongest (r=0.905 for June 2021, r=0.759 for December 2021 for coverage of the first dose of COVID-19 vaccination; r=0.897 for June 2021, r=0.785 for December 2021 for coverage of the complete primary series of COVID-19 vaccination). We also found a moderate correlation between the density of nurses and midwives and COVID-19 vaccination coverage. These findings are mentioned on page 22, lines 375–383. We discuss the significance of these findings in the discussion section (paragraph starting on page 28, line 472), i.e. that this demonstrates the relationship between the health workforce and delivery of vaccination services during a public health emergency. This is particularly important in small island country settings where workforce constraints are even more pronounced. Our findings add to the case for investment in the health workforce, both in quantity and quality to ensure effective and strong vaccination programs during emergencies.

**Changes to manuscript**: Discussion section, page 28, lines 474–478, text added:

This adds to the literature on the importance of having sufficient skilled health professionals to meet the surge requirements of a health emergency, contributing to the case for ongoing investment in building the health workforce. Having an adequate number of skilled health workers enables task shifting and redistributing workers in an emergency response while mitigating the impact on routine services, leading to resilience. *(underlined text is additional)*

Discussion section, page 28–29, lines 487–493 text added:

The need to invest in building the health workforce in SIDS is recognised by target 3c of the Sustainable Development Goals, which is to “substantially increase health financing and the recruitment, development, training and retention of the health workforce in developing countries, especially in least developed countries and small island developing states”.

*Comment 3: Table 5: COVID-19 vaccination coverage at four time points in 2021 and 2022, by economic factors.
I do not think it will be beneficial to do these comparisons. Instead, it would be a good idea to specify the countries in names instead of this classification.*

**Response**: We examined COVID-19 vaccination coverage at four timepoints during the acute phase of the pandemic to examine if associations with independent variables held across all timepoints or if they changed over the 2-year period. Supply constraints earlier in the global rollout (especially in 2021) affected several countries’ ability to scale up their programs. Other factors, such as new COVID-19 variants, changing policies on infection control measures and their impact on the health system would have affected capacity to deliver vaccination programs.

Country-level wealth was extremely influential in access to COVID-19 vaccines during the pandemic, and thus was expected to impact COVID-19 vaccination coverage. This has been demonstrated in other studies, including:

- Nabaggala et al. Int J Equity Health. 2022;21: 147. doi:10.1186/s12939-022-01750-0
- Glassman et al. COVID-19 Vaccine Development and Rollout in Historical Perspective. Washington DC: Center for Global Development Working Paper 607; 2022. Available from: <https://www.cgdev.org/sites/default/files/covid-19-vaccine-development-and-rollout-in-historical-perspective-paper.pdf>

An individual-level analysis would prevent a clear examination of the relationship between COVID-19 vaccination coverage and economic factors in small island developing states. Instead, we have added an appendix that lists which countries fall under each category for the four variables we have included in our analysis.

**Changes to manuscript**: We have included an appendix listing which countries were included in each category for economic factors. Please see appendix S8.

*Comment 4: In total, I do not believe this manuscript will add valuable information to the field.*

**Response**: We thank the reviewer for their feedback. We have expanded the study rationale to more clearly describe our study’s contribution to the field.

**Changes to manuscript**:

Text added to introduction, page 4, lines 96–101, in relation to what the study adds to the literature:

However the study did not examine factors such as the ability to maintain routine immunisation coverage while concurrently implementing COVID-19 vaccination programs (a measure of system resilience), universal health coverage index and infant mortality. These indicators measure different aspects of health and immunisation system performance and including them such analyses provides additional insights into how strengthening routine systems can improve outcomes during an emergency.

Added text to the discussion section:

Page 27–28, lines 456–464, regarding findings related to disruptions to RI and resilience:

The absence of a decline in RI coverage observed in these countries does not imply that there were no disruptions to immunisation services during the pandemic, but more likely that they were able to reorganise and rapidly recover from any disruptions. This ability to respond to and rapidly recover to shocks to the system while responding to the crisis is a defining characteristic of resilient health systems.[41] Resilience is dependent on having a high functioning and adaptive health system, underpinned by equitable access to essential health services and a skilled and adequately sized workforce.[41] Our findings that higher COVID-19 vaccination coverage was correlated with the workforce density and having introduced and sustained newer vaccines reinforce this.

Page 28–29, lines 474–478 and 487–493, regarding findings related to workforce:

This adds to the literature on the importance of having sufficient skilled health professionals to meet the surge requirements of a health emergency, contributing to the case for ongoing investment in building the health workforce. Having an adequate number of skilled health workers enables task shifting and redistributing workers in an emergency response while mitigating the impact on routine services, leading to resilience... The need to invest in building the health workforce in SIDS is recognised by target 3c of the Sustainable Development Goals, which is to “substantially increase health financing and the recruitment, development, training and retention of the health workforce in developing countries, especially in least developed countries and small island developing states”.

Page 30, lines 518–524, regarding findings related to new vaccine introductions:

The benefits of new vaccine introductions such as increased reach of health services following new service delivery mechanisms, increased workforce skill and strengthening adverse event surveillance have been described qualitatively, but are challenging to quantify. Our study provides evidence that the increased flexibility, adaptability and capacity of the health system following new vaccine introductions is linked to achieving higher coverage of vaccination in an emergency scenario.

**Responses to Reviewer 2**

*Overall comment: The manuscript with the title "The association between routine immunisation and COVID-19 vaccination in small island developing states" aimed to understanding the link between routine immunisation (RI) performance and vaccination during an epidemic. The study used multiple data sources (i.e., WHO/UNICEF Joint Reporting Form, World Bank Indicators) to enhance the robustness of the findings. In addition, the study collected information on health system factors, economic indicators, and demographic characteristics, allows for a multifaceted analysis. Below are the comments need to be addressed before consideration for publication.*

**Response**: We thank the reviewer for their thoughtful comments, incorporating their feedback has strengthened our paper’s discussion (see below).

*Comment 1: The study depends primarily on Spearman correlations, which cannot prove causation. The analysis limits the capacity to draw firm conclusions regarding causative pathways. The study lacks extensive statistical modelling (e.g., regression analysis).*

**Response**: We agree that Spearman correlations do not prove causation. However, our intention was to examine the relationship between routine immunisation systems and emergency vaccination. As correlation coefficients describe the strength and direction of an association between variables, we believe these are appropriate statistical measures to achieve our research objective.

We were unable to conduct regression analyses due to limitations in the available data, namely missing data. For example, 13 of 55 SIDS (23.6%) had not reported any data on BCG vaccination coverage for the 5-year period from 2015 to 2019. Fifteen (27.3%) did not report any data on hepatitis B birth dose vaccination coverage for the same period. Data on new vaccine introductions was only available for SIDS who were WHO member states (39/55, 70.9%). Data on various health and immunisation system indicators was also absent, e.g. only 26/55 SIDS (47.3%) had indicated whether they had a system for monitoring adverse events following immunisations.

**Changes to manuscript**: Discussion section edited to acknowledge limitations regarding statistical approach, page 31, lines 541–544, text added:

While this study is unable to draw conclusions about causal associations between routine immunisation systems and COVID-19 vaccination coverage, it does reveal clear links between emergency vaccination and health and immunisation systems in SIDS.

*Comment 2: Inconsistent data from non-WHO member states might limits the findings' applicability to all SIDS.*

**Response**: We agree that inconsistent reporting of data from non-WHO member states may limit our findings’ applicability and have included this limitation in the discussion (page 30, from line 528). We expect country-by-country variation, however we consider our overall conclusions to be applicable to SIDS.

**Changes to the manuscript**: Discussion section, pages 30, lines 529–534, text added:

Inconsistencies in annual reporting and variation in the quality of immunisation coverage data across countries SIDS are potential sources of bias. These biases are mitigated in part through the longstanding standardised process of reporting immunisation coverage estimates through the JRF, a process that has been evaluated and validated.[59] A recent assessment showed that data quality has improved between 2000 and 2019.[60]

*Comment 3: The use of publicly available datasets may result in biases or inaccuracies due to different reporting standards and operation standards.*

**Response**: We acknowledge that there are limitations in the quality of the data (as with most datasets). However, immunisation data collected through the WHO/UNICEF Joint Reporting Form process are highly standardised through a process in place since 1998. All WHO member states report immunisation data annually, which are validated and adjusted by the WHO and UNICEF based on various data sources including surveys. The resulting WUENIC estimate of coverage is the vaccine coverage statistic which we have used in our study. These data have been validated in evaluations. See: Danovaro-Holliday et al. Gates Open Res. 2021;5:77. doi: 10.12688/gatesopenres.13258.1.

**Changes to manuscript**: Edited as above in response to comment #2.

*Comment 4: The study acknowledges the impact of health system resilience but does not delve into other important elements such community trust, vaccine hesitancy, and political pressures.*

**Response**: We acknowledge that our study did not explore all factors which may influence resilience. Our study focuses on measures related to overall immunisation system performance, especially vaccination coverage and introduction of new vaccines which are well-established measures of immunisation system performance (see Patel et al. Glob Health Sci Pract. 2023;11(3):e220055). We have clarified this in our methods section. Data on the factors mentioned by the reviewer is also limited and is not systematically collected, particularly for SIDS. This is noted in the discussion of study limitations (page 31, lines 547–555).

**Changes to manuscript**: Methods section (independent variables: routine immunisation), page 13, lines 212–214:

In this study, we focused on well-established measures of the overall performance of immunisation systems at a national level, namely indicators for vaccination coverage and introducing new vaccines in national immunisation programs.

No additional changes made to other sections – factors mentioned by reviewer are already noted in discussion of study limitations (page 31, lines 547–555).

*Comment 5: Another limitation of the current is that the study does not account for potential lag effects between routine immunization performance and COVID-19 vaccination uptake.*

**Response**: Our intention was to examine the link between the immunisation system performance immediately prior to the COVID-19 pandemic and COVID-19 vaccination coverage. We used 5-year mean annual estimates of coverage which would be a reasonable indication of the state of systems at the time of the pandemic.

**Changes to manuscript**: No changes made.

*Comment 6: The study mentions supply issues. There are other important factors which are not considered in the current study including how geopolitical factors (e.g., vaccine diplomacy, reliance on external donors) impacted vaccine access in SIDS.*

**Response**: We agree with the reviewer that these factors can play a role. However, for the purposes of this study, we wanted to examine the association between COVID-19 vaccination coverage and overall immunisation system performance. Examining all possible factors that may have influenced COVID-19 vaccination coverage was out of scope. Data on all these factors are also not consistently or systematically reported for SIDS. Noting the considerable shifts during the pandemic, these were difficult to measure with no baseline data.

We have noted the additional factors raised by the reviewer in the limitations section.

**Changes to manuscript**: Discussion section, page 31, lines 554–555, text added:

Other factors, such as policies related to mandatory vaccination requirements for work or travel, geopolitical factors including vaccine diplomacy, and the role and influence of external donors and global health partner organisations could have also influenced COVID-19 vaccine uptake. *(underlined text is additional)*

*Comment 7: The study depends on countries with good reporting system for Routine Immunisation.*

**Response**: We acknowledge that the quality of immunisation coverage data likely varies across countries, and we mention this in our discussion of study limitations. However, the Joint Reporting Form process builds in methods to adjust for these variations. The method of coverage estimation is described in our response to point #3. While not perfect, this is the best available data on vaccination coverage.

**Changes to manuscript**: No additional changes made (changes to address this point were made in relation to comment #3 above).

*Comment 8: The focus of this study is on aggregate country-level statistics does not consider within-country differences, which may be considerable in SIDS.*

**Response**: Our study aimed to look at performance at the national level, and thus within-country variations in performance were out of scope for our analysis. We agree that there may be within-country variations in performance. Subnational level data for SIDS can be problematic to interpret given the very small country sizes, highly mobile populations and challenges with variations in small numbers reflecting as large differences in proportions, so we have focused on the national level. The challenges with subnational level coverage data were raised by experts from the Pacific Islands in a study we conducted. See: Patel et al. PLOS Glob Public Health. 2024; doi: 10.1101/2024.03.12.24304182

**Changes to manuscript**: Discussion, page 30, lines 534–538, text added:

We also did not examine inequities in vaccination coverage, for COVID-19 or routine vaccinations, within countries, due to the lack of publicly available data, poor quality of subnational level data and challenges in interpreting variations in coverage proportions in countries with small and highly mobile populations.[61]

*Comment 9: The study shall provide more specific recommendation based on the findings, particularly in resource-limited areas.*

**Response**: Thank you for this suggestion. We have included clearer recommendations in our discussion.

**Changes to manuscript**: Conclusions section, page 32, lines 569–573, text added:

We recommend that countries continue to invest in their health workforce including those working in immunisation. Increasing efforts to introduce new vaccines into national immunisation schedules, including implementing new service delivery mechanisms to reach populations beyond the first year of life, can increase adaptability and thus place countries in a better position to deploy epidemic and pandemic vaccination programs.
