# Supplementary material for: The association between routine immunisation and COVID-19 vaccination in small Island developing states
Source: PLoS One. 2025 Jul 8;20(7):e0317327. doi: 10.1371/journal.pone.0317327 (PMC12237071; doi:10.1371/journal.pone.0317327)
Supplement: Attachment — Submitted filename: Responses to reviewer comments_final_2025.05.14.docx [file pone.0317327.s013.docx]

**The association between routine immunisation and COVID-19 vaccination in small island developing states**

**Response to reviewers’ comments**

We thank the reviewers for the comments on our paper and for the opportunity to address them. We have clarified certain aspects of our paper and included additional details in the manuscript in response. Below, we provide responses to each of the reviewers’ comments and line number references where we have made changes, or point to where existing text in the manuscript addresses comments.

We note that there were no comments from reviewers #2 and #3.

**Responses to reviewer 1**

*1. Table 1 does not add any extra information to your manuscript, the authors could add a brief of how the analysis made to the method section.*

Thank you for this comment. Table 1 provides the details on the independent variables included in the study and information on data sources. Our manuscript includes a summary of this information in the methods in the sections describing independent variables (see lines 219-261) and the data sources (lines 179-201). However, we feel the detail in the table is necessary for clarity and transparency of our methods. Including all this information in text form would be tedious for readers. We have included the table so that our methods are sufficiently detailed for another researcher to replicate them. We have made some additional clarifications to the manuscript text on data sources.

Added text underlined (lines 179-201):

We extracted the following publicly available data (Table 1):

- Monthly COVID-19 vaccination coverage data for 2021 and 2022 from COVID-19 Vaccination Information Hub, collated from the WHO Joint Reporting Form (JRF) COVID-19 vaccination module.[30]
- WHO/UNICEF estimates of national immunisation coverage (WUENIC) data for the years 2015 to 2022, collated from countries annually via the JRF process. Data were sourced from the WHO Immunization Data Portal, including data on RI coverage, new vaccine introduction and immunisation system performance.[31] Data for RI coverage were not consistently reported for non-WHO member states, and were unavailable for new vaccine introduction status and immunisation system performance measures through the WUENIC system. Indicators on dropout of routine immunisation coverage and disruptions to routine immunisation coverage during the COVID-19 pandemic were calculated based on WUENIC estimates.
- World Bank Indicators data for health system performance (e.g. health worker density and childhood mortality rates) and country-level demographic characteristics (e.g. country population size).
- Country income level and development status classifications as defined by the World Bank and United Nations Development Programme, respectively.

Table 1 provides further details on data sources for independent variables. All data were extracted in April 2024. As all data were aggregated at the country-level and obtained from publicly available sources, ethical approval was not required.

*2. Could you give more details about the income levels in Table 2, how they have been classified to Low, Lower-middle, Upper-middle, and Other*

Country income level categories are based on the World Bank’s classifications using the World Bank Atlas Method, which uses Gross National Income per capita to categories countries. Their methodology is detailed in this link: <https://datahelpdesk.worldbank.org/knowledgebase/articles/906519-world-bank-country-and-lending-groups>

We have clarified this in our manuscript text (line 196-197) and the footnote to Table 1 (line 205-206).

Edited text underlined:

Line 196-197: Country income level and development status classifications as defined by the World Bank and United Nations Development Programme, respectively.

Line 212-213: Country income level categories are defined by Gross National Income (GNI) per capita based on the World Bank’s classifications using the World Bank Atlas Method.

*3. Rewrite the conclusion section in your manuscript, indicates what is the main finding or conclusion from your study that summaries the result section*

We have edited the conclusion section to summarise the main findings and conclusions from the study.

Added text, line 581-583: In conclusion, we found that countries that achieved higher COVID-19 vaccination coverage also sustained pre-pandemic RI coverage during the pandemic, had introduced newer vaccines into their national immunisation schedules, and had higher workforce density.

**Responses to reviewer 4**

*The ability of developing countries to adapt quickly and withstand changes is often limited by the lack of infrastructure, personnel, and adequate equipment in the health sector. I believe that this study is not only relevant, but essential, and should be approached from a critical-scientific perspective in order to promote substantial improvements and provide a solid foundation for future health policy recommendations and studies. Below, I present my comments with the aim of strengthening its structure and presentation:*

Thank you for this feedback.

*Abstract
Introduction:
1.- It would be beneficial to include a more detailed characterization of the study population. Additionally, include a brief contextualization of the level of public trust in vaccines, public health policies, and the current state of the logistics system.*

We have added some additional details as requested.

Additional text added:

Lines 113-116: SIDS vary in their income level classifications from low through to high income, but are all considered to be vulnerable to systemic shocks due to social, economic, environmental and infrastructural challenges and limited resources.[9]

Lines 132-135: Additionally, they face higher freight costs to transport vaccines due to their remote geography. Vaccine distribution to the point-of-service is complicated by the need to maintain cold chain in warm climates and transporting vaccines by air and boat especially to remote outer lying islands.

Lines 139-150: Policies regarding border closures, quarantine protocols and vaccine mandates varied, as did decision-making and emergency response coordination structures.[15,20] For example in the Pacific, country-level responses were complemented by the WHO’s wider Pacific Joint Incident Management Team partner coordination meetings.[20] In the Caribbean, the Caribbean Public Health Agency Caribbean Regulatory System collaborated with the WHO and Pan American Health Organization to expedite regulatory approvals of COVID-19 vaccines.[23] Acceptance of COVID-19 vaccination was also a challenge in some SIDS, with surveys in Papua New Guinea, Haiti and Dominican Republic indicating only 27.6%, 43.2% and 68.8% of individuals, respectively, intended to receive a COVID-19 vaccine.[24,25] Another survey of healthcare workers found broad (>90%) acceptance of vaccines in general, but hesitancy to receive COVID-19 vaccines among 23% of respondents.[26]

*Discussion:
1.- Generally, in a scientific research, the discussion should be written in the third person to achieve a formal style and maintain objectivity.*

Thank you for this feedback. We have reviewed the manuscript and believe that the language used in the discussion is appropriate. The discussion is written in a formal academic voice, and we have maintained consistency throughout. The style and tone are similar to other recently published articles in PLoS One, e.g.:

- Brehon et al. PLoS One, 2025; 20(5): e0322911 <https://doi.org/10.1371/journal.pone.0322911>
- Hiranburana et al. PLoS One, 2025; 20(4): e0317940. <https://doi.org/10.1371/journal.pone.0317940>
- Acharya et al. PLoS One, 2025; 20(4): e0322031. <https://doi.org/10.1371/journal.pone.0322031>

We appreciate preferences for writing style vary and will work with the editorial team to finalise the manuscript.

*2.- The paragraph:
"The finding that SIDS that did not experience declines in RI coverage during the pandemic achieved high COVID-19 vaccination coverage aligns with the principles of a “strong” health system being a resilient one. The absence of a decline in RI coverage observed in these countries does not imply that there were no disruptions to immunisation services during the pandemic, but more likely that they were able to reorganise and rapidly recover from any disruptions... "
Is too long and repetitive, with too many ideas competing for attention, which weakens the overall message.*

We have revised this paragraph and tried to simplify the message.

Revised text underlined, lines 464-488:

We found that SIDS that did not experience declines in RI coverage during the pandemic also achieved high COVID-19 vaccination coverage. This supports the principle that a “strong” health system is a resilient one. The absence of a decline in RI coverage observed in these countries does not imply that there were no disruptions to immunisation services during the pandemic. Rather, it is more likely that they were able to reorganise and rapidly recover from any disruptions. This ability to rapidly recover from shocks to the system while responding to the crisis is a defining characteristic of resilient health systems.[46]

Resilience is dependent on having a high functioning and adaptive health system, underpinned by equitable access to essential health services and a skilled and adequately sized workforce.[46] While higher COVID-19 vaccination coverage was correlated with the workforce density and having introduced and sustained newer vaccines, we did not find an association between universal health coverage and COVID-19 vaccination coverage. This is contrary to a recent study that demonstrated countries with higher universal health coverage index scores had higher rates of COVID-19 vaccination.[47] Our findings might reflect intensified international development partner efforts in response to COVID-19 especially in lower income countries, or the public response to COVID-19 control measures, such as requirements for vaccination certificates for travel.[48]

*3.- It would be possible to conduct multivariate analysis on a smaller sample with complete data, which could improve the analysis and address one of the study's limitations.*

A rule of thumb in multivariate regression is that there should be at least 10 observations for every independent variable included in the model. The degree of missing data meant that any multivariate analysis we conducted could not have included more than 3 independent variables. For example, 13 of 55 SIDS (23.6%) had not reported any data on BCG vaccination coverage for the 5-year period from 2015 to 2019. Fifteen (27.3%) did not report any data on hepatitis B birth dose vaccination coverage for the same period. Data on new vaccine introductions was only available for SIDS who were WHO member states (39/55, 70.9%). As we would have needed to control for country-level statistics like income level and country size, the small sample size meant we could not to explore the relationship between RI variables in any further depth.

*Conclusions:
1.-It would be beneficial to investigate whether SIDS cultural or context-specific factors within the sample may have influenced these results.*

We agree these could have affected COVID-19 vaccination uptake. However, this study primarily examined the association between COVID-19 vaccination coverage and overall immunisation system performance. Examining all possible factors that may have influenced COVID-19 vaccination coverage was out of scope. Data on all these factors are also not consistently or systematically reported for SIDS. We have noted this as a limitation in our discussion section, please see lines 568-577 copied below.

Existing text: Additional factors that could be examined include service delivery and accessibility, vaccine acceptance and demand, and cold chain capacity, which were beyond the scope of this study. Cultural and political factors also have a role, with multiple studies reporting that trust in government was strongly associated with higher, faster and earlier uptake of COVID-19 vaccination and other outcomes during the pandemic including COVID-19 mortality.[8,67,68] Other factors, such as policies related to mandatory vaccination requirements for work or travel, geopolitical factors including vaccine diplomacy, and the role and influence of external donors and global health partner organisations could have also influenced COVID-19 vaccine uptake.

**Responses to reviewer 5**

*A review report of the manuscript entitled “The association between routine immunisation and COVID-19 vaccination in small island developing states”*

*- (lines 49-50) The current sentence fails to clearly explain the statistical tools used in the data analysis. Please revise it by mentioning on which program the analysis was done and the statistical power used to determine the significance.*

We have edited the sentence to clarify our statistical approach.

Edited text underlined, line 49-50: We calculated Spearman correlation coefficients (r) with p-values (p<0.05 considered significant) and 95% confidence intervals for continuous variables and mean COVID-19 vaccination coverage by categorical variables.

*- (lines 55-61) Please unify the statistical report written on each sentence. There’s still disparity in the current way of reporting, especially in the usage of “r”, which should be applied uniformly.*

We have edited the abstract to clarify our statistical approach, specifically our use of correlation coefficients. In the results section of the abstract, we have checked that we have consistently reported the r value and p-value for the relevant statistics.

Edited text underlined, line 49-50: We calculated Spearman correlation coefficients (r) with p-values (p<0.05 considered significant) and 95% confidence intervals for continuous variables and mean COVID-19 vaccination coverage by categorical variables.

*- (line 75) The introduction fails to include relevant literature. The authors should improve their literature review by including more relevant published papers. Additionally, besides summarizing previous work on the topic, the authors should link their approach with previous studies by highlighting the state-of-the-art and gap in the literature.*

Our paper examines the relationship between routine immunisation systems and emergency vaccination, specifically COVID-19 vaccination. Only one other study has quantitatively examined this relationship in a multi-country analysis to date, which we have summarised in lines 96-102. We have reviewed our introduction section to ensure the relevant existing literature is cited.

*- Please give a bit of overview on what COVID-19 is in the introduction. For example, “COVID-19 is induced by a positive-sense single-stranded RNA virus known as severe acute respiratory syndrome coronavirus 2 (SARS-CoV-2). Individuals afflicted with COVID-19 may exhibit minor symptoms such as fever, cough, headache, and sputum production, which can escalate to more serious complications, including acute respiratory distress syndrome (ARDS) and mortality.” This info can be cited from a study by Duta et al. (2023) entitled “Essential oils for COVID-19 management: A systematic review of randomized controlled trials”.*

We have added an overview of COVID-19.

Added underlined text, line 91-95: The COVID-19 pandemic provides an opportunity to apply a health systems lens to examine the link between RI performance and emergency vaccination. COVID-19, caused by the severe acute respiratory syndrome coronavirus 2 (SARS-CoV-2), can cause severe respiratory disease requiring hospitalisation and critical care such as ventilation, and can be fatal.[7] Its high transmissibility threatened to quickly overwhelmed health systems in the first wave of infection, leading to intense efforts to develop and deploy an effective vaccine. A recent analysis of COVID-19 vaccination coverage and immunisation program maturity found that COVID-19 vaccination coverage was 14-16% higher in countries with an adult seasonal influenza vaccination program.

*- (line 143) What were the exclusion criteria of this study?*

We have added a statement to make our exclusion criteria clear.

Added text (line 176-177): We only excluded SIDS for which COVID-19 vaccination coverage was not publicly reported.

*- (lines 160-163) How did the authors deal with inconsistently reported RI data measured through the WUENIC system?*

We included all available data for each specific comparison to maximise the amount of data we could use, i.e. analyses of COVID-19 vaccination coverage by new vaccine introduction were limited to the 39 countries for which data were reported. Denominators were based on the number of countries for which data were available for. This is summarised in our manuscript in the methods section, see lines 280-290. The limitations of the inconsistently reported data are included in the discussion section, see lines 549-555.

Existing text on how we managed missing data (lines 280-290): We included data from SIDS that reported COVID-19 vaccination coverage data and were publicly available, excluding SIDS from analyses where data for specific independent variables were unavailable. In analyses with RI coverage, we used the mean of available coverage values (i.e. the denominator was the number of years for which data were available). We used 5-year mean annual immunisation coverage for RI rather than individual year estimates to account for year-to-year variation in coverage and mitigate against the impact of an anomalous year for a specific country in our analysis. We excluded SIDS from specific analyses only if they did not have any coverage values reported for a specific vaccine over the 5-year period. We limited the analyses by new vaccine introduction variables and immunisation system performance variables to WHO member states only (n=39), as data for non-member states were not available through WUENIC.

Existing text on limitations of inconsistently reported data (lines 549-555): Our analysis was limited by the lack of publicly available coverage data for all routinely-administered vaccines in SIDS, particularly for non-WHO member states. Inconsistencies in annual reporting and variation in the quality of immunisation coverage data across SIDS are potential sources of bias. These biases are mitigated in part through the longstanding standardised process of reporting immunisation coverage estimates through the JRF, a process that has been evaluated and validated.[64] A recent assessment showed that data quality has improved between 2000 and 2019.[65]

*- (line 188) It is unclear what kind of first vaccine dose the authors mean in this phrase: “The primary outcomes of interest were coverage of: A) the first dose…”*

We have edited the sentence to improve clarity.

Edited text (line 219-221): The primary outcomes of interest were coverage of: A) the first dose of COVID-19 vaccination, and B) a complete primary course of COVID-19 vaccination. We examined coverage at four timepoints in the first two years of the global roll-out…

*- Authors are suggested to proofread the manuscript after addressing all comments to avoid any typological, grammatical, and lingual mistakes and errors. For example, there’s an extra space on line 279 and a misspelling on line 280; repetition on lines 299 and 361.*

Thank you for identifying the typographical error in the footnotes for Table 1, we have rectified this. We have also reviewed the full manuscript as requested.

Please note that the repetition of asterisks in the footnotes for Table 3 and 4, i.e. notations of “**”, are not repetition but are used to denote different levels statistical significance. In Table 3, one asterisk denotes a significance of p<0.05, while two asterisks denotes p<0.01. For Table 4, three asterisks denotes <0.001. We would be open to using a different style for these notations if requested by the editors, following acceptance of the article.

*- (lines 467-468) This sentence “Least developed countries, with weaker health systems,[54] had lower COVID-19 vaccination coverage throughout the study period.” will be more significant if it’s also supported by another relevant study. For example, a study by Rosiello et al. (2021) entitled “Acceptance of COVID-19 vaccination at different hypothetical efficacy and safety levels in ten countries in Asia, Africa, and South America”*

Thank you for this suggestion, however this statement is a reflection of findings from our study. We have now clarified this.

Edited text, line 517: In our study, least developed countries, with weaker health systems,[59] had lower COVID-19 vaccination coverage throughout the study period.

**Responses to reviewer 6**

*Thank you for giving me the opportunity to review your manuscript and addressing the comments of the previous reviewers. I have no further questions or comments.*

Thank you to the reviewer for their consideration of our manuscript.

**Responses to reviewer 7**

*Author have answered all the discussion given before and provide a better explanation. Some added information are also helping to explain the main focus of this study.*

Thank you to the reviewer for their consideration of our manuscript.
